# Warming inhibits increases in vegetation net primary productivity despite greening in India

**DOI:** 10.1038/s41598-023-48614-3

**Published:** 2023-12-03

**Authors:** Ripan Das, Rajiv Kumar Chaturvedi, Adrija Roy, Subhankar Karmakar, Subimal Ghosh

**Affiliations:** 1 Interdisciplinary Program in Climate Studies, Indian Institute of Technology Bombay, Powai, Mumbai, 400 076 India; 2 Department of Humanities and Social Sciences, Birla Institute of Technology and Science-Goa Campus, Zuarinagar, India; 3 Department of Civil Engineering, Indian Institute of Technology Bombay, Powai, Mumbai, 400 076 India; 4 Environmental Science and Engineering Department, Indian Institute of Technology Bombay, Powai, Mumbai, 400 076 India

**Keywords:** Climate sciences, Ecology

## Abstract

India is the second-highest contributor to the post-2000 global greening. However, with satellite data, here we show that this 18.51% increase in Leaf Area Index (LAI) during 2001–2019 fails to translate into increased carbon uptake due to warming constraints. Our analysis further shows 6.19% decrease in Net Primary Productivity (NPP) during 2001–2019 over the temporally consistent forests in India despite 6.75% increase in LAI. We identify hotspots of statistically significant decreasing trends in NPP over the key forested regions of Northeast India, Peninsular India, and the Western Ghats. Together, these areas contribute to more than 31% of the NPP of India (1274.8 TgC.year^−1^). These three regions are also the warming hotspots in India. Granger Causality analysis confirms that temperature causes the changes in net-photosynthesis of vegetation. Decreasing photosynthesis and stable respiration, above a threshold temperature, over these regions, as seen in observations, are the key reasons behind the declining NPP. Our analysis shows that warming has already started affecting carbon uptake in Indian forests and calls for improved climate resilient forest management practices in a warming world.

## Introduction

Terrestrial vegetation serves as a CO_2_ sink in the global carbon cycle, absorbing 31% of anthropogenically emitted CO_2_^1^. During photosynthesis, it absorbs atmospheric CO_2_ and converts it to a higher energy form, carbohydrate, which it then stores as biomass^2^. Recent satellite observations have concluded that the Earth's green cover has increased significantly in the last two decades^3–6^. The 'greening' is generally measured with the Leaf Area Index (LAI), computed with one-sided leaf area for broadleaf canopies and half of the needle surface area for coniferous canopies^7^. This global greening trend is majorly contributed by CO_2_ fertilization effect^3,4^ but at a regional scale, the dominant factors are climate (for example: the rising temperature in the high latitudes)^3,4^ and Land use and Land Cover (LULC)^3,4,8,9^.

This global greening intuitively enhances vegetation productivity and hence, increases the carbon sink potential, which is defined by the capacity of the ecosystem to absorb and store atmospheric CO_2_ through photosynthesis. However, the recently published observed^10^ and model-driven^11^ studies have not found a proportional increase in vegetation productivity at a global scale. Gross Primary Productivity (GPP) is one of the key indicators for measuring vegetation productivity. GPP is defined as the amount of CO_2_ captured by the plants in unit time during the time of photosynthesis. GPP has increased only 0.08%, whereas the global green cover has increased about 0.23% during the period 2000–2015. The lower increase in GPP is attributed to the atmospheric moisture stress globally^11^. Net Primary Productivity (NPP) is another important indicator for determining vegetation productivity. NPP is the difference between GPP and autotrophic respiration. There exists inconsistency in the global trends between NPP and GPP arising due to increasing autotrophic respiration^10,12^. Temperature and precipitation are reported to be the two most important climatic factors that have control over vegetation productivity^13–17^. Zhang et al.^13^ hypothesized that increasing temperature drives global NPP decrease, despite a stable GPP for the terrestrial ecosystem. There is increasing evidence that climate extremes such as droughts and storms inhibit the expected increase of vegetation carbon uptake^15,18–20^. Temperature^21^, total water storage^22^, and soil moisture^23^ also play a major role in global carbon uptake by terrestrial vegetation and thus in the interannual variations of the Carbon Growth Rate (The growth rate of atmospheric CO_2_ concentration in the atmosphere^22^).

Among the tropical regions, India is the second-highest contributor to post-2000 global greening after China^5^. However, it is not clear if such a greening resulted in an overall increase in primary productivity and thus in carbon uptake potential, given the impacts of changing climate on the vegetation. The majority of the studies also do not include the recent period and have considered pre-2010 years. Observational analysis with Advanced Very High Resolution Radiometer (AVHRR) data for the period 1982–2006 showed a 3.9% increasing trend of NPP/ decade in India, which has been attributed to CO_2_ fertilization^24^. Other studies^25,26^ with observational data and model output showed that precipitation is the major driver behind NPP variations. Nayak et al.^26^ also estimated an increase of NPP at 0.005 PgC year^-2^ during 1981–2006. Model-driven study^27^ showed an increasing trend of NPP during 1901–2010 at 1.2–1.7 PgC.year^−2^ due to elevated CO_2_, LULC changes, and nitrogen deposition. A similar increasing trend of NPP was also reported for the period 2001–2006 with AVHRR and MODIS data^28^. The key research question addressed in this study is the translational potential of increasing green cover in India in recent decades to an overall increase in primary productivity in a changing environment with a warming trend. To address this question, we have used MODIS quality-controlled satellite data of LAI, GPP, NPP, and Net Photosynthesis (PSNnet) (details in the Method section) for the entire period of 2001–2019. We performed the trend analysis and found a slight decreasing trend of NPP, and stable GPP during the 21^st^ Century despite the increase in LAI. The results are contrary to the findings from earlier studies^24,26–28^ that date back up to 2000s data. Notably, there are spatial variations in the NPP trend with the biggest contributing regions of NPP in India suffering from a larger decreasing trend. We further investigated key factors (LULC and climate) influencing spatial variability in NPP trends using different statistical analysis (trend, linear regression, and Pearson’s correlation). Our analysis revealed that regions with significantly decreasing NPP trends have experienced the largest warming during the study period. We further used the non-linear kernel regression method (details in the Method section) to study the temperature response of vegetation productivity over these regions. We observed that photosynthesis dropped and respiration became stable above a threshold temperature over these regions. We have also used the Granger causality analysis to further ascertain the climate controls on changing PSNnet (and subsequently, NPP). Granger causality analysis confirmed that the decrease in NPP in India is attributable to the recent warming of the twenty-first century.

## Results

### Trends of LAI, GPP and NPP

**Figure 1:**
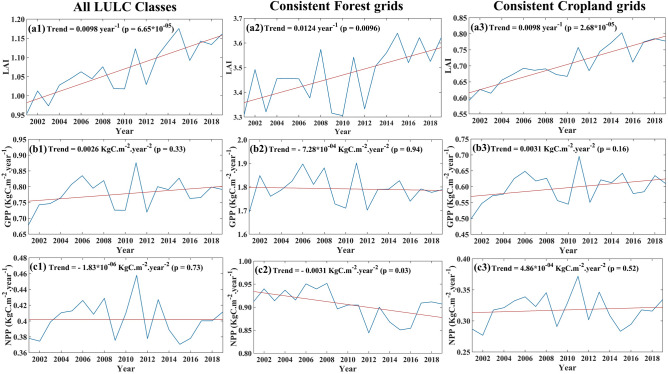
Trend Inconsistencies between Greening and Carbon Uptake in India: The time series of LAI (**a1**), GPP (**b1**) and NPP (**c1**) over India during the period 2001–2019. We have selected the grids over India where the land use was consistently forest and cropland during 2001–2019. The time series of LAI (**a2**), GPP (**b2**), and NPP (**c2**) over consistent forest grids in India. The time-series of LAI (**a3**), GPP (**b3**), and NPP (**c3**) over consistent cropland grids in India. Figure 1 is generated using MATLAB 2023a software (https://www.mathworks.com/?s_tid=mlh_gn_logo).

Figure 1a1–c1 presents the annual time series of LAI, GPP, and NPP over India. We find that the LAI over India has increased steadily (statistically significant) in the twenty-first century, which is consistent with the findings from previous studies^5,29^. The annual time series of GPP and NPP have not shown any statistically significant trend despite this greening; the NPP showed a slight (not statistically significant) decreasing trend. NPP, GPP, and LAI products are obtained from MODIS radiometric information. There is a high chance that these datasets are affected by covariability. Extensive ground truth verifications are also not possible for India due to the limited number of flux tower locations having long-term observed datasets. Furthermore, the MODIS products are derived data and do not consider increasing productivity due to carbon fertilization^30^. We have used FLUXCOM GPP and Net Ecosystem Exchange (NEE) data products to ensure our results are robust. Figure S1 shows the climatology of MODIS GPP and FLUXCOM GPP over the Indian landmass. Both products show high photosynthesis during the last half of the monsoon, August and September. However, the climatology between MODIS and FLUXCOM has substantial differences during the pre-monsoon and winter seasons. The values from MODIS maintain high GPP starting from the post-monsoon till February, while the FLUXCOM GPP drops significantly. It is expected that the GPP in India during post-monsoon should be maintained due to high radiations and high soil moisture from monsoon recharge. The same may continue in the winter, a cropping season in India known as Rabi season. MODIS GPP dips during pre-monsoon summer because it is a dry, non-cropping season. The FLUXCOM GPP climatology shows an increase from February–March, which is unexpected. MODIS GPP is calculated using the MOD17 algorithm^31^, taking the input of remote sensing and meteorological data. MOD17 algorithm was created based on Monteith^32^ radiation conversion efficiency concept. FLUXCOM GPP is generated by machine learning methods with the help of remote sensing data and meteorological data^33,34^. A previous study has mentioned that the FLUXCOM product largely captures GPP's response to instantaneous climate variability alone and does not capture the effect of the current vegetation state^35^. None of these data products are observations, and they have different methodological approaches, which resulted in differences in climatology.

We plotted trends for FLUXCOM GPP and FLUXCOM NEE in Fig. S2. The FLUXCOM GPP (Fig. S2a) shows a statistically non-significant decreasing trend, and FLUXCOM NEE (Fig. S2b) shows a statistically significant increasing trend over the Indian landmass. These are consistent with the results obtained from MODIS GPP and NPP trends. Both FLUXCOM GPP and NEE products showed that the greening in India has not been translated to vegetation productivity.

Land use change^11^ or climatic impacts^11,12^ could have potentially inhibited the growth of GPP and NPP in India. The annual time series of both forests and croplands area show steadily increasing trends over the last two decades (Fig. S3). During this period, forest cover has increased by 0.25%, while cropland has increased by 0.65%, as shown in Fig. S4a. This increase in forest mainly came at the expense of savannas, and for cropland the major contributor is grassland, as indicated in Fig. S4b,c. Hence, land use change is apparently not the reason behind the non-increasing NPP at a country scale. In Fig. S5, we represent the LULC map of India for the years 2001(Fig. S5a) and 2019 (Fig. S5b). We use changing classes of LULC to distinguish between anthropogenic and climate drivers in our current analysis. We select the grid points, which were forestland consistently during the period 2001–2019 and present the time series of LAI, GPP, and NPP (Fig. 1a2,b2,c2, respectively) spatially averaged over those grids. The LAI over the forestland shows a statistically significant increase. However, the GPP over the forestland shows a slight decrease (not significant), and the NPP shows a statistically significant decreasing trend. In India, forests are generally untouched by anthropogenic management interventions like irrigation, fertilizer use etc. One of the strong possibilities could be the impacts of changing climate on the forest ecosystem, which resulted in a decrease in primary productivity. Similar plots for the croplands show increased LAI (Fig. 1a3), and stable (no statistically significant trend) GPP and NPP (Fig. 1b3,c3, respectively). The croplands are heavily managed, and hence, the natural impacts of climatic factors on croplands may be mitigated/altered by human interventions. This finding helps us to distinguish between anthropogenic and climate drives. The vegetation productivity in croplands is primarily driven by anthropogenic factors, whereas climate plays a significant role in productivity in forestlands.

**Figure 2:**
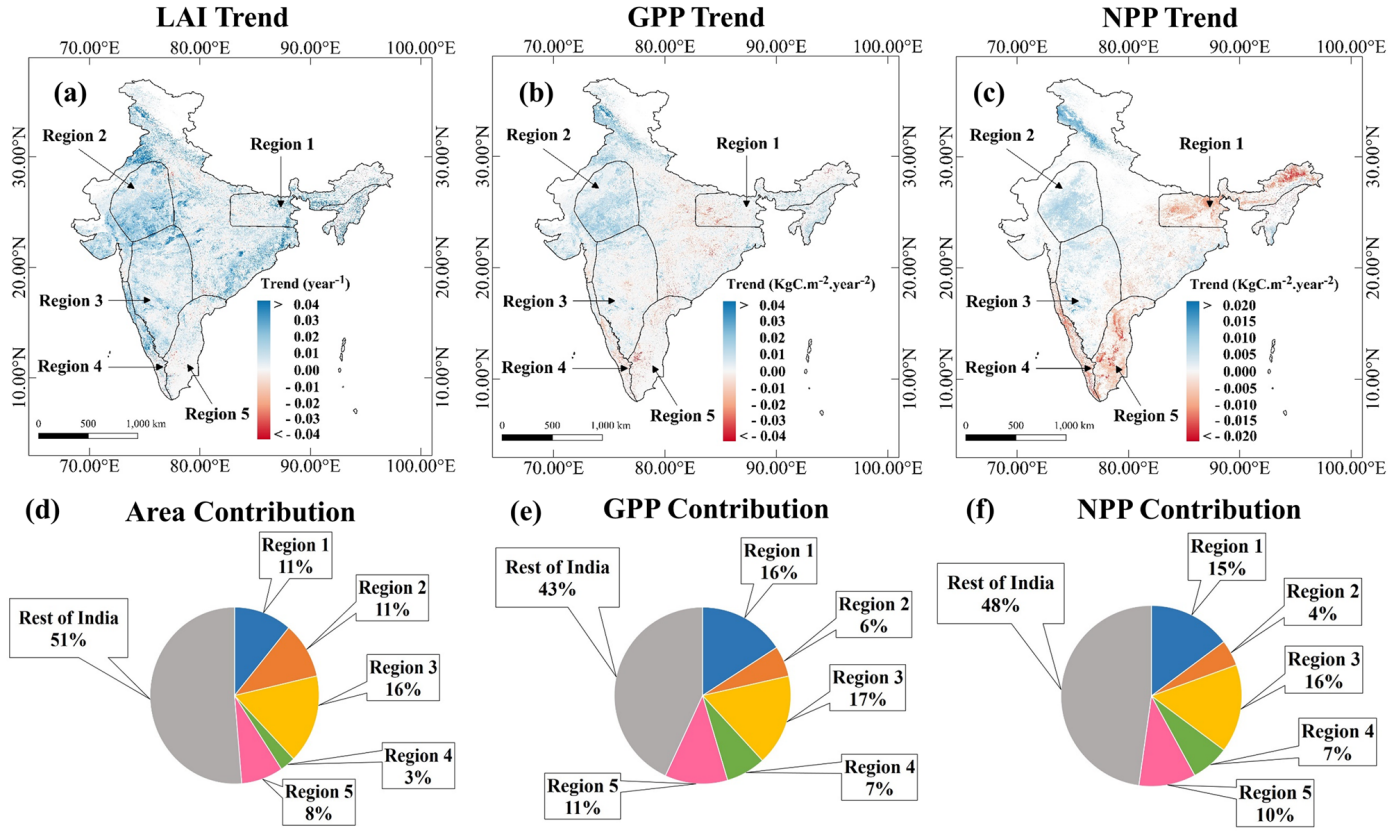
Regional Trends: Trends of LAI (**a**), GPP (**b**), and NPP (**c**) during the period 2001–2019 at a statistically significant level of 0.1, based on the sign of trends, 5 Regions were selected, Region 1: Northeast, Region 2: Northwest Arid, Region 3: Central Peninsular, Region 4: The Western Ghats, and Region 5: East Coast Peninsular. Region 1, 4, and 5 have decreasing trends of NPP, while 2 and 3 have increasing trends of NPP. The area contributions (**d**) from regions 1, 4, and 5 to the total country area is 22%, whereas the GPP and NPP contributions are 34% and 32% of the national estimates, ((**d**) and (**e**)), respectively. Figure 2a–c are generated using QGIS 3.16 software (https://www.qgis.org/en/site/forusers/download.html) and Fig. 2d,2e and 2f. are generated using Microsoft PowerPoint Office 365 software (https://www.microsoft.com/en-in/microsoft-365/powerpoint).

The spatial map of trends in MODIS LAI shows a widespread increase all over India (Fig. 2a). However, the trends of GPP and NPP from the MODIS have strong spatial variations. Many of the regions over Northeast India and Peninsular India have a statistically significant decreasing trend of GPP, as evident from Fig. 2b. The regions with decreasing NPP are more prominent over the Northeast and coastal peninsular India. We selected five regions based on the direction of NPP trends observed in this study (Fig. 2c). Depending on the spatial extents of these trend patterns we identified the regions to further investigate the drivers (LULC/Climate) of these regional trend patterns. We identified Northeast India as Region 1, The Western Ghats as Region 4, and the East Coast Peninsula as Region 5, as these regions exhibited decreasing NPP trends despite increasing LAI. On the other hand, the Northwest Arid Region was classified as Region 2, and the Central Peninsular Region as Region 3, and they showed positive NPP trends. We further standardized the MODIS LAI, MODIS GPP, and MODIS NPP datasets and calculated their trends. The standardization of these three variables places them on the same scale and allows us to better understand their evaluations in different regions. The trend maps for the standardized MODIS LAI, MODIS GPP, and MODIS NPP datasets are shown in Fig. S6a–c respectively. The results of Fig. S6a–c are consistent with those of Fig. 2a–c. We have also plotted the spatial variations in the trends of FLUXCOM GPP and FLUXCOM NEE (Fig. S7a,b respectively). The FLUXCOM GPP (Fig. S7a) trends are not statistically significant in most regions, except for Region 2. In Region 2, we observed a few grids showing a negative trend in GPP. However, MODIS GPP (Fig. 2b) shows a positive trend in Region 2. It is noteworthy that none of these data products are observations. The opposite trend in GPP over Region 2, might be attributed because of their different methodological approaches, including different meteorological input data sources. However, we found strong increasing trends of NEE (Fig. S7b) (signifying declining ecosystem carbon uptake) almost everywhere in India. NEE increasing trends are stronger in regions 1, 4, and 5 compared to other regions. MODIS NPP also shows significant decreasing trends in these regions (1, 4, and 5). These spatial patterns probably signify that vegetation carbon uptake over regions 1, 4, and 5 has been affected over the last two decades compared to the rest of India. Regions 1, 4, and 5 contribute to only 22% of India's area; however, their contribution to India's NPP and GPP are 34% and 32% (as per the MODIS product), respectively (Fig. 2d–f). It is noteworthy that regions 1 and 4 contain the forests of Northeast India and the Western Ghats, which are among the world's eight hottest hotspots of biodiversity^36^ and account for significant fraction of India’s forest area and resulting forest carbon uptake. Now onward we are performing our analysis with the MODIS GPP and NPP, considering their spatial trend consistency with the FLUXCOM NEE product in regions 1, 4, and 5.

We have applied a data quality control filter to the LAI and GPP datasets to remove cloud cover and bad pixels. Our analysis revealed that regions 1 and 4 exhibit a lower count of good pixels on an annual scale (Fig. S8a). Notably, these regions also display a decreasing trend in GPP/NPP (Fig. 2b,c). The decline in vegetation productivity trends in these areas may be influenced by the lower pixel count. To further investigate this, we have presented the good pixel count for non-monsoon (JFMAMOND) (Fig. S8b), and monsoon (JJAS) (Fig. S8c) periods. During the monsoon season (Fig. S8c) we observed a lower pixel count from Region 1 and Region 4. This lower pixel count in monsoon is attributed to lower pixel count at the annual scale. However, during non-monsoon season (Fig. S8b) both these regions (1 and 4) maintain a good pixel count.

We also plotted the trends of LAI, GPP, and PSNnet for both the non-monsoon (Fig. S9a–c) and monsoon (Fig. S9d–f) seasons. LAI exhibits an increasing trend in both seasons across most regions of the country (Fig. S9a,d). During the non-monsoon period, our analysis shows that GPP exhibits a significant increasing trend in Region 2. Notably, there are no statistically significant changes in vegetation productivity in Region 3 due to the absence of monsoon rainfall. However, regions 1, 4, and 5 experience decreasing trends in GPP (Fig. S9b). During the monsoon period, we observe an increasing trend in GPP over regions 2 and 3. The increasing trend in LAI/GPP in these regions (2 and 3) may be associated with increased monsoon rainfall. However, regions 1, 4, and 5 exhibit negative trends (Fig S9e). The seasonal analysis of LAI and GPP trends is consistent with the annual trend. Since NPP is only available annually, we plotted the PSNnet trend (difference between GPP and Leaf and Fine Root Maintenance Respiration) in Fig. S9c,f. PSNnet also shows a negative trend in regions 1, 4, and 5 in both seasons, which is consistent with the annual NPP trend (Fig. 2c). The trends for GPP and LAI obtained from the annual data are consistent with those obtained for the non-monsoon season when the good pixel counts are high across the country. These seasonal analyses confirm that our main finding of a decreasing NPP trend despite greening is not influenced by lower sample sizes.

In Fig. S10, we separately analyzed trends of LAI, GPP, and PSNnet for the winter season (DJF). In the winter season, we observed an increase in LAI only over Region 2, no significant trends in LAI were observed in other regions (Fig. S10a). Region 2 is a cropland-dominant area (Fig. S5) and contributed to increased LAI through increased Rabi season cropping. GPP also exhibited an increasing trend over Region 2 during the winter season. In regions 4 and 5, we observed a weak decreasing trend in GPP (Fig. S10b). By comparing the seasonal trends between monsoon and winter, we found that the trends in GPP during the monsoon season are higher compared to those for the winter season (Except for Region 2). The key reason is changing monsoon patterns, which need further investigation. In both the monsoon (Fig. S9f) and winter season (Fig. S10c), regions 4 and 5 also display a negative trend in PSNnet. These seasonal trends in PSNnet strongly suggest that the net atmospheric carbon uptakes over these regions are consistently affected during both the seasons, monsoon and winter.

**Figure 3:**
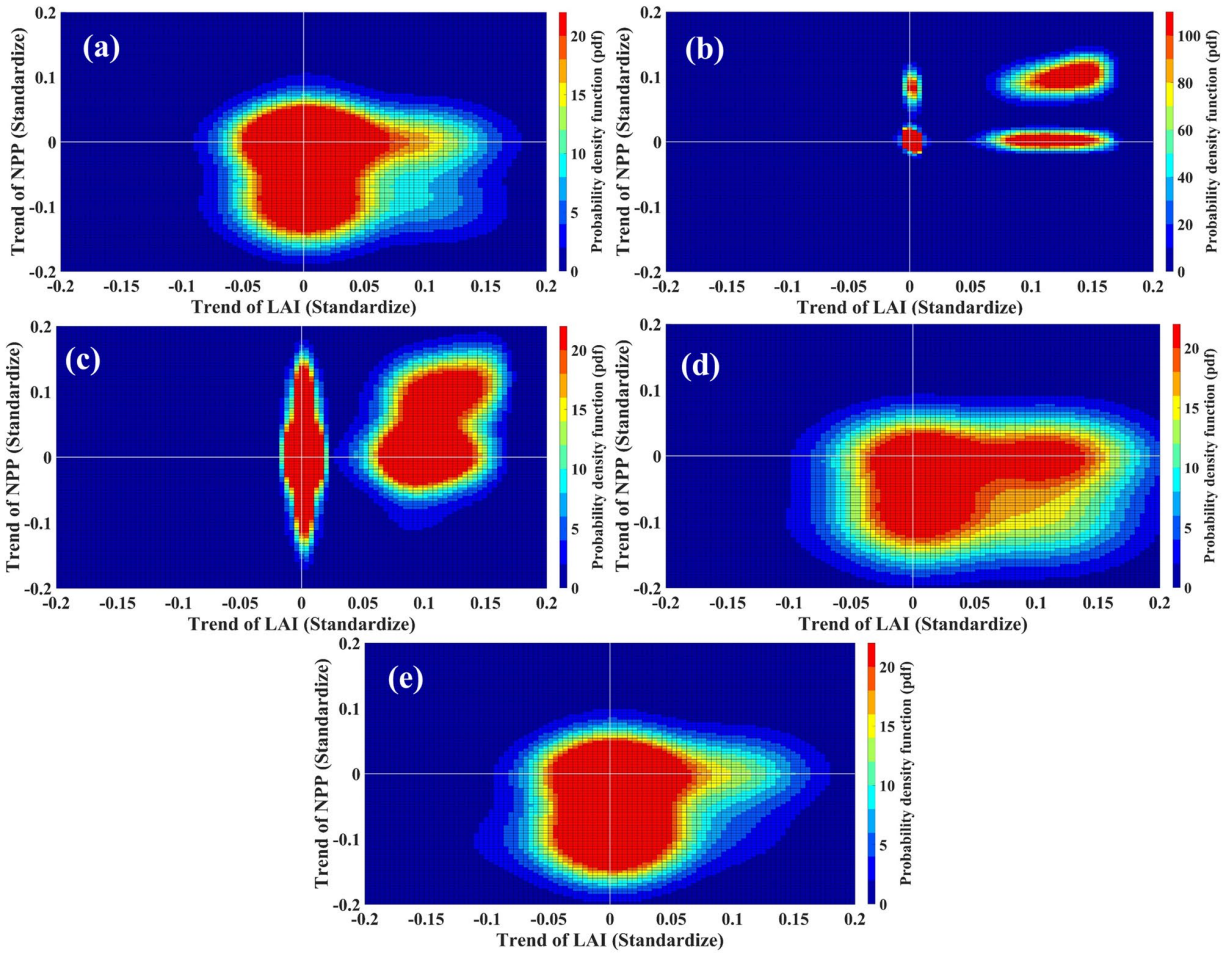
Divergence of Trend (density): Probability density function (pdf) of the trends of standardized LAI and NPP trends using bivariate kernel density estimate for Region 1: Northeast (**a**), Region 2: Northwest Arid (**b**), Region 3: Central Peninsular (**c**), Region 4: The Western Ghats (**d**) and Region 5: East Coast Peninsular (**e**) for the period 2001–2019. Figure 3 is generated using MATLAB 2023a software (https://www.mathworks.com/?s_tid=mlh_gn_logo).

We visually observed that in regions 1, 4 and 5, the LAI trends diverge from the NPP trend; to demonstrate this statistically, in Fig. 3, we generated probability density functions (pdf) of standardized LAI and NPP trends using a bivariate kernel density estimate for each region. We also produced a scatter plot of standardized LAI and NPP trends (Fig. S11). Each point in the sample for generating pdfs (Fig. 3) and in the scatter plot (Fig. S11) represents a single grid. We plotted the LAI (Standardized) trend on the x-axis and the trend of NPP (Standardized) on the y-axis. The density of LAI trend pixels is higher in the first and fourth quadrants in all five regions, signifying widespread greening over these five regions. We also found a trend divergence between LAI and NPP in regions 1 (Figs. 3a, S11a), 4 (Figs. 3d, S11d), and 5 (Figs. 3e, S11e). Pixel density (Fig. 3) was higher in the fourth quadrant of these three regions, where the LAI trend value is positive and the NPP trend value is negative. These findings are consistent with the trend maps (Figs. 2, S6). These plots demonstrate more quantitatively that the LAI trend diverges from the NPP trend during the study period in India's Northeast (Region 1), Western Ghats (Region 4), and East Coast Peninsula (Region 5).

We zoom in the regions 1, 4, and 5, which have statistically significant decreasing trends in NPP, in Figs. S12, S13, and S14. For Region 1, the Northeast India, the increase in the LAI is widespread (Fig. S12a), though there are a few grids showing a decrease in LAI at the eastern side (Fig. 2a). Such decreases are due to recent deforestation at isolated locations in the eastern states, such as Meghalaya, Arunachal Pradesh, and Nagaland^37^. However, the decreasing trends in NPP (Fig. S12c) are widespread over Northeast India, where there has been greening in most regions during 2001–2019. The GPP also has a decreasing trend except for central Northeast India. The Western Ghats region (Region4) also has (Figs. 2, S13) decreasing GPP and NPP despite increasing LAI. Recent study^38^ and the State of forest report in India^37^ confirm that there is effectively no deforestation in the Western Ghats in the 21st Century. There are a few spots with decreasing LAI in the southern Western Ghats in the state of Kerala. This is probably due to declining paddy cultivation in Kerala that resulted from changing the agricultural practices to home gardening^39^. However, such isolated declining spots cannot result in a widespread decrease in NPP. In Region 5, there are a few patches of declining LAI (Fig. 2a) and in those patches, GPP and NPP have also declined (Fig. 2b,c). However, similar to regions 1 and 4, the GPP and NPP decrease is widespread (Fig. S14b,c) in Region 5 as well.

In Fig. S15, we plotted the standardized time series of forestland area, cropland area, LAI, and NPP for all five regions. We found that for Region 1, the changes in NPP are not consistent with changes in forest land and cropland (Fig. S15a,b). The same is true for regions 4 (Fig. S15g,h), and 5 (Fig. S15i,j). Therefore, land use and land cover changes cannot account for the divergence in trend between LAI and NPP over these regions. However, for regions 2 (Fig. S15c,d) and 3 (Fig. S15e,f) there is a consistency, which shows the likely impacts of LULC changes on vegetation greenness and productivity. Detailed discussions of Fig. S15 have been added to Supplementary Text 1.

We linearly regressed NPP as a function of LAI (NPP = f (LAI)) and looked into the correlation between NPP and LAI to perform a more detailed analysis of the annual variation. In regions 1 (Fig. S16a) and 4 (Fig. S16d), we found that the goodness of fit (R^2^) value was very low and statistically insignificant at level 0.1. Additionally, Fig. S17 shows a negative correlation between LAI and NPP over the majority of the regions in 1 and 4. However, most regions 2, 3, and 5 show a positive correlation (Fig. S17) between LAI and NPP. As a result, we can infer that the relationship between LAI and NPP is stronger in the regions (2 and 3) where there is no trend divergence between LAI and NPP. In contrast, the relationship between LAI and NPP is negative in the regions (1 and 4) where LAI and NPP trends diverge. Interestingly, the LAI over Region 5 also does not show a strong positive trend or greening (Fig. S14a). Hence, the negative NPP does not result in a negative correlation in Region 5, as observed in regions 1 and 4.

### Climate controls on the changing NPP/GPP

**Figure 4:**
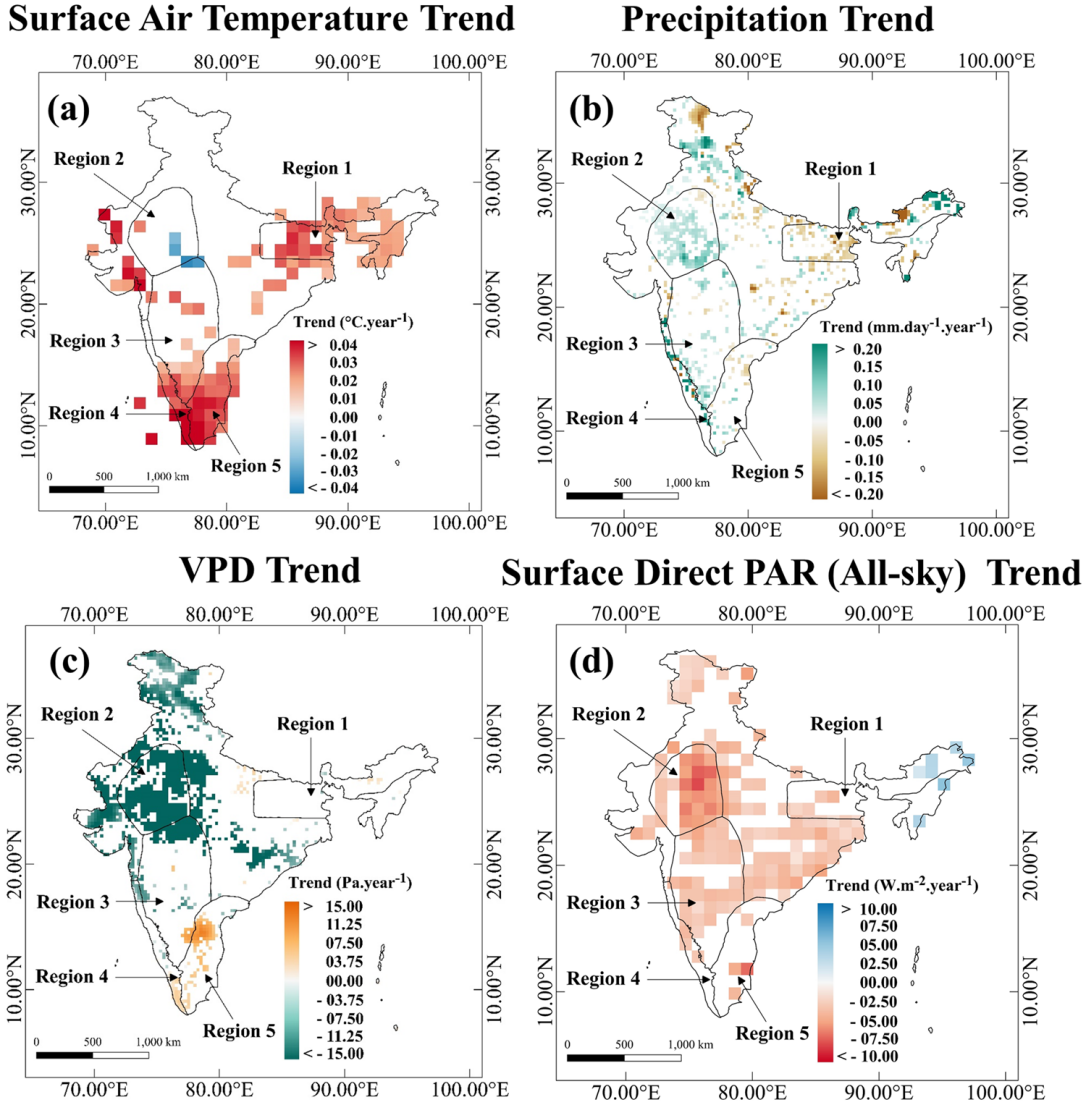
Climatic Trends: Trend of IMD Gridded Surface Air Temperature (**a**), IMD Gridded Precipitation (**b**), Vapour Pressure Deficit (calculated using ERA5 reanalysis relative humidity and temperature data) (**c**), and CERES Surface Direct PAR (All-sky) (**d**) in India for the period 2001–2019. The Figures only show statistically significant trends at 0.1 level. Figure 4 is generated using QGIS 3.16 software (https://www.qgis.org/en/site/forusers/download.html).

To understand the climate connections to the declining NPP, we first present the trends of surface air temperature, precipitation, vapour pressure deficit and surface direct photosynthetic active radiation (Fig. 4). Warming is prominently visible (Fig. 4a) only over regions 1, 4, and 5, pointing out a strong association with the declining GPP and NPP. Regions 1 and 5 experienced a mixed trend of precipitation (Fig. 4b), whereas the precipitation has increased over Region 4. There is a lack of similarity or spatial pattern match between the signs of precipitation trends and GPP/NPP trends. A strong warming trend has been observed in regions 1, 4 and 5 but the Vapour Pressure Deficit (VPD) trends in these regions (Fig. 4c) are not significant. A high increase in temperature does not necessarily result in a high increase in VPD due to the role played by atmospheric humidity. In Fig. S18a,b, we plotted the trends of ERA5 temperature and relative humidity, datasets respectively. We observe almost no change in relative humidity (Fig. S18b) over these regions (1, 4 and 5). This could be the reason behind a high-temperature increase and a moderate VPD increase in regions 1, 4, and 5. The VPD shows an increasing trend in some parts over regions 4 and 5 (Fig. 4c) that observed declining NPP/GPP. The northern part of Region 4 shows a decreasing trend in VPD, and the changes in GPP/NPP are also slow in the same Region. In regions 2, and 3 (except the southern part) there are no statistically significant changes in temperature. There is indeed a decrease in VPD, which could be associated with high atmospheric moisture (Fig. S18b) and may also be reflected in terms of monsoon precipitation. A significant decreasing trend in Photosynthetic Active Radiation (PAR) values (Fig. 4d) has been observed in regions 2, and 3. However, both GPP and NPP shows an increasing trend over regions 2 and 3 (Figs. 2, S6). We found a few decreasing grids of PAR in the regions (1, 4, and 5) where NPP decreased but the trend patterns are inconsistent. Notably, a previous study^29^ also found that MODIS GPP was increasing in some areas where decrease in PAR was observed. The negative correlation observed between GPP and solar radiation in pervious study^29^ could be attributed to the opposing trends of GPP and radiation.

In Fig. S19, we've plotted the seasonal trends of temperature and precipitation to demonstrate how they influence LAI and vegetation productivity differently during the dry and wet seasons. We observe significant warming trends over regions 4 and 5 during the non-monsoon (dry) season (Fig. S19a). Trend in precipitation is negative at a few grids in regions 4 and 5. However, the sign of precipitation trends is not spatially consistent within the individual regions (Fig. S19b). Precipitation trends also cannot explain the trend in PSNnet during the non-monsoon season due to the absence of major rainfall. We found that decreasing trend of PSNnet (Fig. S9c) coincides with warming trends of Region 4 and 5, during the non-monsoon season.

During the monsoon (wet) season, we observe a strong warming trend over most of the areas in Region 1 (Fig. S19c). Regions 4 and 5 also observed warming but the fraction of area of significant warming is less compared to that of the non-monsoon period. PSNnet shows a strong negative trend over regions 1, 4, and 5 (Fig. S9f). However, over regions 4 and 5, the negative trend is weaker in the monsoon compared to the non-monsoon season. In Region 2, we observed a consistent increasing trend in Precipitation (Fig. S19d) GPP and PSNnet also show a significant increase trend over the Region during the Monsoon season over Region 2.

This seasonal analysis show the contribution of temperature and precipitation during both the non-monsoon and monsoon periods. This seasonal pattern of temperature reflects the negative impact of warming on net vegetation productivity, which is consistent with the observed annual trends of temperature and NPP. Given that temperature and precipitation are reported to be the two most important climatic factors that have control over vegetation productivity^13–17^, our seasonal analysis were focused exclusively on these two variables.

**Figure 5:**
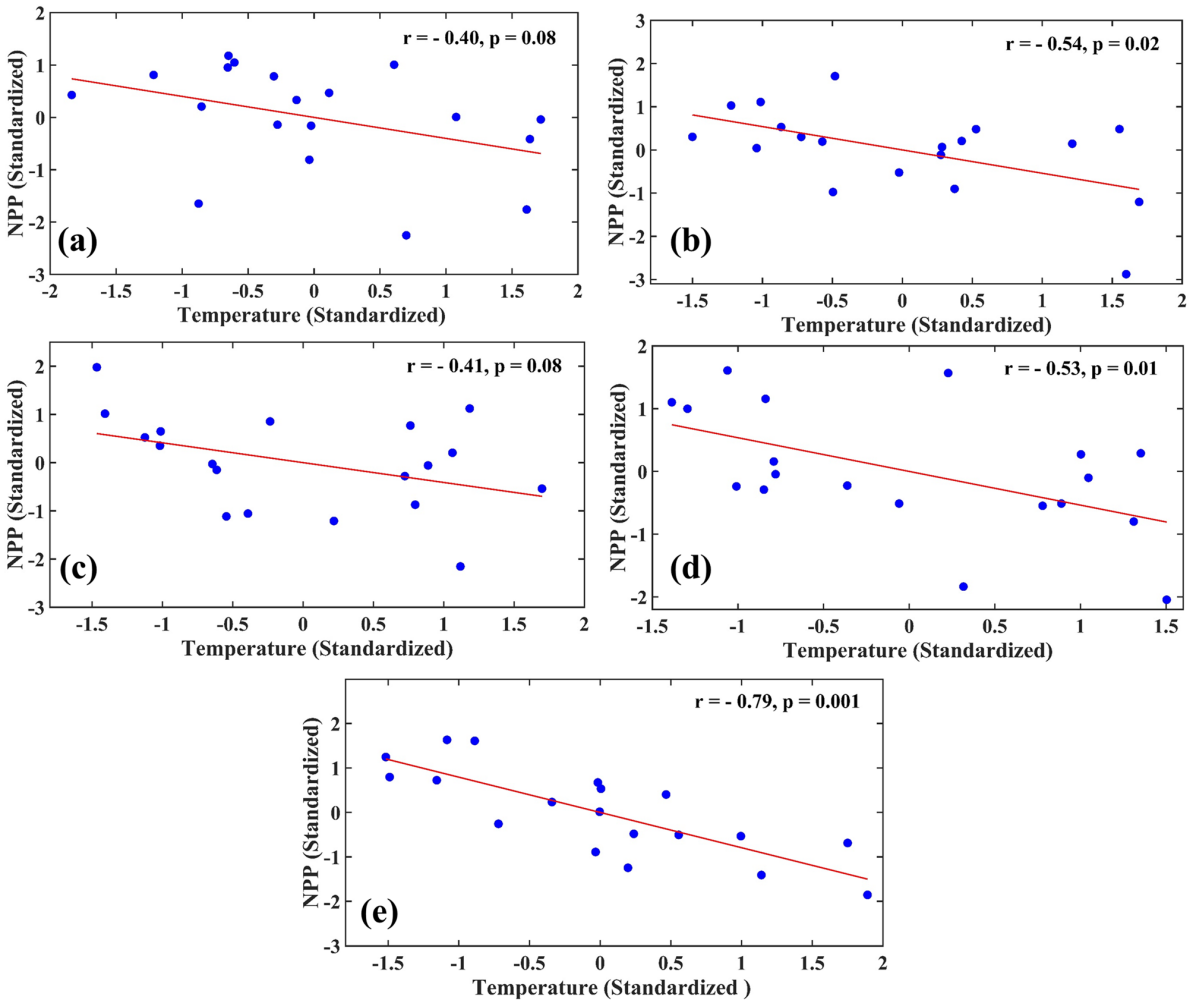
Impacts of Temperature on NPP: Scatter plot between annual mean temperature (standardized) and NPP (standardized) for Region 1 (**a**), 2 (**b**), 3 (**c**), 4 (**d**) and 5 (**e**) during the period 2001–2019. Figure 5 is generated using MATLAB 2023a software (https://www.mathworks.com/?s_tid=mlh_gn_logo).

There is a spatial resemblance between India's warming hotspots (regions where a significant warming trend has been observed) and the regions with declining NPP, indicating a strong temperature control on vegetation productivity. In Fig. 5, we have shown the scatter plots between annual mean temperature (Standardized) and NPP (Standardized) for all five regions. We have also calculated Pearson correlation coefficient (r) between temperature and NPP for these regions. Temperature exhibits a significant negative correlation with NPP in all five regions. A previous study also found that NPP and temperature have a negative correlation in most places in the country^40^. For a quantitative understanding of the role of temperature on the diverging trend of NPP and LAI, we linearly regressed NPP as a function of LAI (NPP = f (LAI)) and calculated the trend in the residuals for each grid (Fig. S20). We computed the residuals as follows, Residual = NPP (observed)—NPP (simulated by regression); NPP (observed) represents the actual observed values of NPP. The NPP (simulated by regression) provides the values of NPP simulated by the linear regression model with annual LAI as a regressor. The residual trend is decreasing in nature in regions (1, 4, and 5). In these regions (1, 4 and 5), we also observed a significant warming trend (Fig. 4a). We also show the scatter plots between temperature and the residuals of linear regression (NPP against LAI) and calculated Pearson correlation coefficient (r) in Fig. S21a–e. We found that the unexplained (by LAI) components of NPP are negatively correlated with the temperature for all regions. These plots (Figs. 5, S20, S21) demonstrate the negative impacts of warming on the NPP.

**Figure 6:**
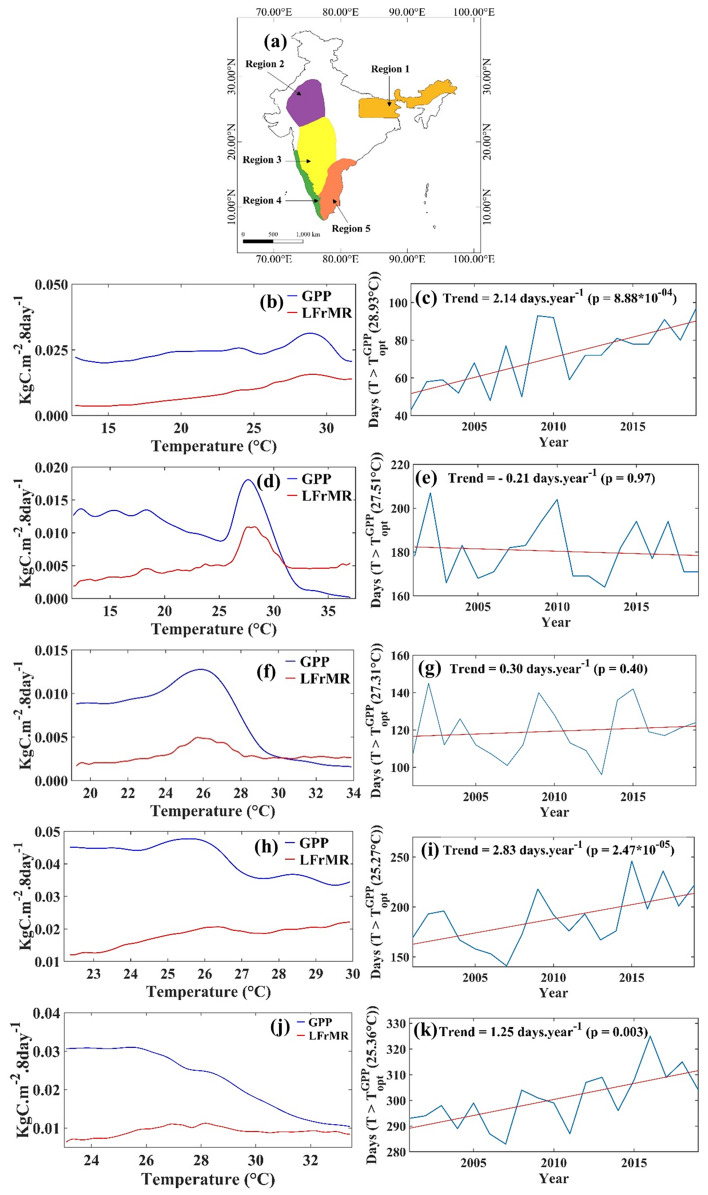
Warming Impacts the Vegetation Productivity: Map showing Regions (**a**), Variation of Gross Primary Productivity (GPP) and Leaf and Fine root Maintenance respiration (LFrMR) to temperature for Regions 1 (**b**), 2 (**d**), 3 (**f**), 4 (**h**), and 5 (**j**). The optimum temperature is the temperature at which GPP reaches the maximum. The GPP drops above this temperature. The trend of the number of days in a year exceeding optimum temperature for Regions 1 (**c**), 2 (**e**), 3 (**g**), 4 (**i**), and 5 (**k**) during 2001–2019. Figure 6a is generated using QGIS 3.16 software (https://www.qgis.org/en/site/forusers/download.html) and Fig. 6b–k are generated in MATLAB 2023a software (https://www.mathworks.com/?s_tid=mlh_gn_logo).

The GPP generally increases with temperature till an optimum value. When the temperature crosses the optimum value, GPP declines^41^ while respiration rises^42^, leading to decrease in NPP^43,44^. Tropical forests typically have the aforementioned characteristics, with a decrease of 9.1 mega grams of carbon uptake per hectare per degree Celsius warming in the mean daily maximum temperature in the warmest month^45^. We plot the variations of GPP in the 5 selected regions with temperature in Fig. 6. The curves in the subplots are the smoothed variations as obtained using nonlinear kernel regression^46^. From these curves, we obtained the temperature (termed as optimum temperature) value where GPP reaches the maximum, and from there, it starts dropping. We observe a significant difference in optimum temperatures between regions. In Fig. S5, we have presented the LULC map of India, which highlights the diversity in land cover classes across various regions. The variability in optimum temperatures among the regions can be attributed to the different vegetation classes found in each region. Notably, different vegetation classes respond differently to temperature; thus, the variation in GPP with temperature varies across different regions. We calculated Leaf and Fine root Maintenance respiration (LFrMR) by taking the difference between GPP and PSNnet and using them in Fig. 6. For regions 1, 4, and 5, the LFrMR are either stable or increased beyond the optimum temperature. Hence, with warming above the optimum temperature, the net Photosynthesis (PSNnet, the difference between GPP and LFrMR) should decrease in these regions with a subsequent decline of NPP. We also investigated the equations used to calculate the MODIS NPP and solved them analytically (Supplementary Text 2) to look into the temperature response of leaf and fine root maintenance respiration rates individually and collectively (Fig. S22). We found that the rate of change in Leaf Maintenance Respiration (Leaf MR) with temperature drops after 25° C (Fig. S22a); however, it remains positive, suggesting an increase in Leaf MR with the temperature. The rate of change in fine root maintenance respiration steadily increases with temperature (Fig. S22b) and the total rate of change in leaf and fine root maintenance respiration becomes stable after 30° C (Fig. S22c). The results are very similar to those obtained from Fig. 6b,d,f,h,j showing consistency.

The time series and the trend of the number of days in a year above the optimum temperature (T^GPP^_opt_) for the regions are presented in Fig. 6c,e,g,i,k. For regions 1, 4, and 5, there are statistically significant increasing trends in the number of days above optimum temperature (T^GPP^_opt_). These trends, along with low photosynthesis above optimum temperature, result in low net carbon uptake by the vegetation. Interestingly, for regions 2 and 3, the LFrMR drops after the optimum temperature; however, the drops are not as high as the drops in GPP. Region 2 is India's warmest and region with high aridity, and probably the vegetation is already adapted to a warm environment. Notably, in Region 5, majority of days throughout the year experience the optimum temperature, yet the vegetation does not seem to adapt. Region 2 is arid, and Region 5 is humid. In Region 2, RH has increased significantly (Fig. S23a); hence VPD has decreased (Fig. 4c), which is favourable for the plants. The magnitude of GPP's optimum temperature is higher for Region 2 than Region 5 (Fig. 6). Notably, in Region 2, we found that LFrMR drops with optimum temperature and becomes stable, indicating that vegetation has likely adapted to this warm and arid environment. In contrast, Region 5 maintains relatively stable RH levels (Fig. S23b) but experiences a significant increase in temperature (Fig. 4a). Hence, in Region 5, GPP drops, but LFrMR remains stable. The response of vegetation productivity patterns to temperature and adaptive capacity varies across different land cover types. This is most likely the reason behind the vegetation not adapting in Region 5 despite majority of days experiencing the optimum temperature. For regions 2 and 3, the number of days exceeding the optimum temperature does not have a trend. Hence, the decline in NPP is not observed in these regions.

**Figure 7:**
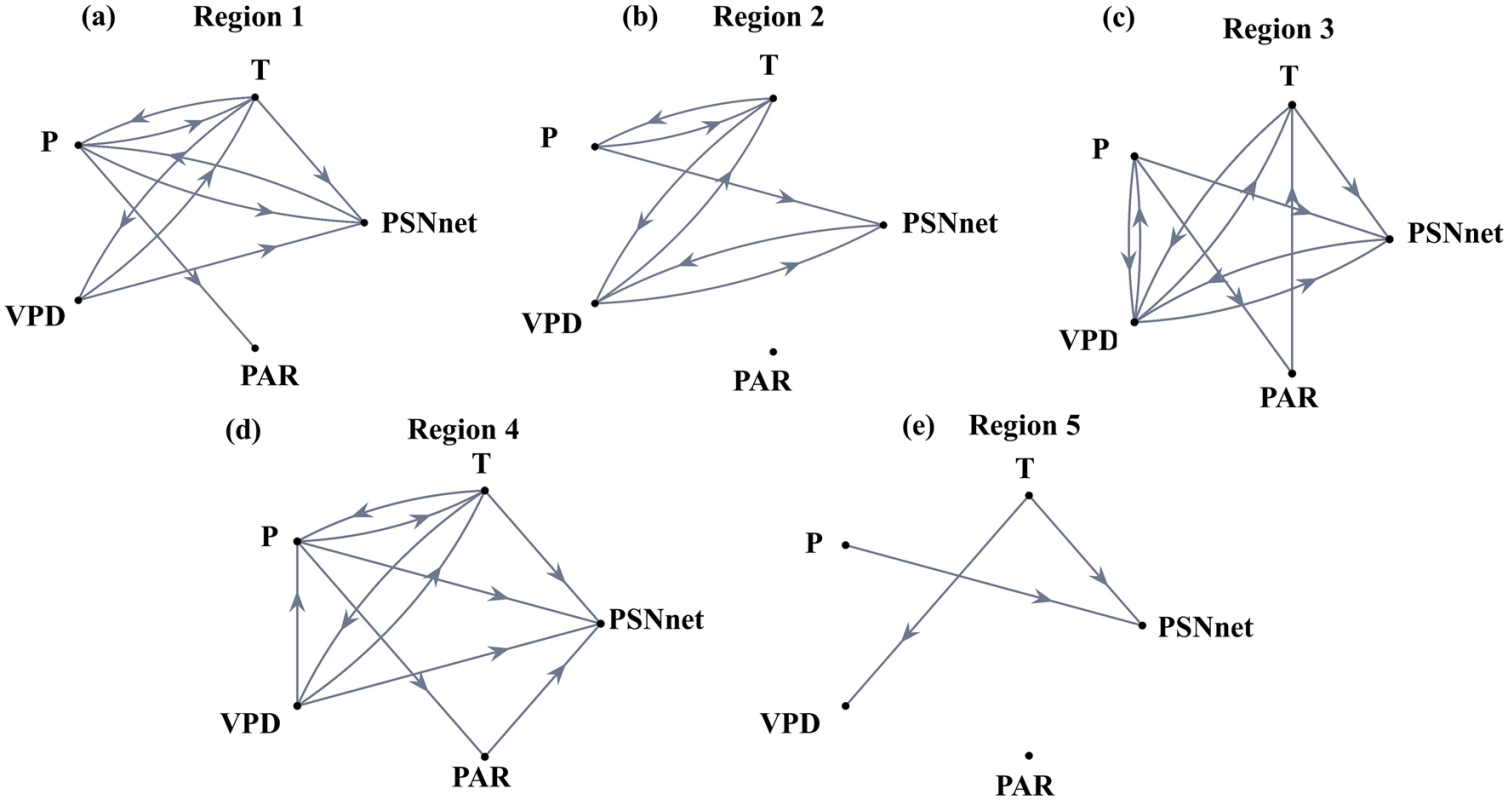
Causal Network between Climate and Productivity Variables: The causal network derived from Granger causality at the Regions 1 (**a**), 2 (**b**), 3 (**c**), 4 (**d**), 5 (**e**). The variables used are Precipitation (P), Temperature (T), Vapour Pressure Deficit (VPD), Photosynthetic Active Radiation (PAR), and net Photosynthesis (PSNnet) for the period 2001–2019. The causal links are shown from source to sink using an arrow at a statistically significance level of 0.05. Figure 7 is generated using MATLAB 2023a software (https://www.mathworks.com/?s_tid=mlh_gn_logo).

We use the Granger causality approach to further ascertain the climate controls on changing PSNnet (and subsequently, NPP). The results are presented in Fig. 7 for all the 5 Regions. Except for Region 2, all the other regions, shown causal links from temperature to PSNnet. For Region 2, temperature causes vapour pressure deficit that subsequently causes PSNnet. Hence, for all the regions, the causal links from temperature to PSNnet are established. Figures 2, 3, 4, 5, 6 and 7 collectively show that the warming impacts the net carbon uptake by vegetation with a declining trend of NPP over regions 1, 4, and 5. It should be noted that precipitation has a causal connection to PSNnet as expected over the monsoon region of India. However, there is no consistent trend similarity between Precipitation and GPP or NPP. Hence, the causal link from Precipitation to PSNnet in Fig. 7 presents the covariation of the variables at seasonal or intra-seasonal scale as reported by Valsala et al.^25^. For regions 3 and 4, we observed causal links from PAR to PSNnet (Fig. 7). In other regions, no links from PAR to PSNnet were observed. To further confirm the causal connections, we plot the causal networks with GPP in Fig. S24, similar to Fig. 7. They show that for all the regions, the temperature has causal connections either to GPP hence affects the PSNnet and NPP. Using Granger causality, we may identify the causal factors; however, quantifying contributions from a causal variable to an impact variable is difficult. Furthermore, all the meteorological variables are connected, with the possibility of multiple confounding factor(s). Hence, we did not attempt to quantify the exact causal contribution from temperature to productivity. This is one of the limitations of the present causal analysis and probably a generalized limitation of any widely used causal discovery methods. In Supplementary Table 1 and 2, we have mentioned mentioning the significance level (*p*-value) for each causal link presented in the causal diagrams for each region. However, this “*p*-value” does not quantify the casual strength, but the statistical significance.

## Discussion and concluding remarks

Recent decades have experienced increasing atmospheric CO_2_ concentration and widespread warming around the globe. India is not an exception. Increased warming resulted in an increase in water vapour carrying capacity and a subsequent increase in the vapour pressure deficit. Stomatal conductance reduces under increased VPD and transpiration increases till a VPD threshold^47,48^. Both of these processes lead to reduced photosynthesis and these impacts have been observed globally^47,48^. Global studies showed that the VPD increased by 0.0017 ± 0.0001 kPa year^−1^ (Data: CRU) during 1999–2015. GPP decreased by 0.23 ± 0.09 PgC year^−1^ and 0.31 ± 0.11 PgC year^−1^ since 1999, as per the EC-LUE and MODIS models, respectively^49^. On the contrary, model-driven studies show improved Water Use Efficiency (WUE)^50–52^ and vegetation productivity under increased CO_2_ concentration due to carbon fertilization^50,51,53^. The scientific question remains if carbon fertilization can make up for the reduced productivity of the vegetation due to warming and the increased VPD. A synthesis study performed in the IPCC Assessment Report 6, Working Group 1, Chapter 5^1^, and Chapter 11^54^ showed a high confidence that the vegetation carbon sink will be less efficient at a very high warming level.

There are a few limitations in our study:

1. The primary outcomes of this study are based on MODIS datasets. Satellite products like MODIS do not consider carbon fertilization. On the other hand, the models are still inadequate to consider the physiological response of plants to the increased VPD. The opposing impacts of both the factors and the resulting uncertainty in projections are more pronounced at a lower warming level. The literature agrees that high VPD impacts will dominate globally at a higher warming level over carbon fertilization due to multiple direct and indirect effects^1^. The lack of ground-truth data availability in India is one of the major bottlenecks in performing a detailed analysis of vegetation productivity trends and responses to increased VPD and carbon fertilization.
2. We used MODIS satellite data in our study, which has limitations associated with the quality and accuracy of data. MODIS Products are sensitive to the sky conditions, and data quality is affected by the cloud cover^55^. In situ data does not have such limitations, but cannot spatially cover a large Region due to the sparse network and non-availability. However, they can be used to ensure the quality of satellite data. Unfortunately, in India, long-time in-situ data is not openly available. Thus, we had to rely on satellite products.
3. In this current study, we performed multiple independent statistical tests to show that warming is the key factor behind decreasing productivity. The statistical tests identify linear associations between the variables. We did not perform any non-linear causal analysis, which is a limitation of this current study. If we use nonlinear causal analysis, such as PCMCI with information theory^56,57^ or transfer entropy^58,59^, it is not possible to state which sign of change in causal variable X results in the direction of changes in impacted variable Y. Such sign information is important for our present analysis since the hypothesis is that positive temperature changes result in negative productivity changes. Hence, we restrict ourselves to linear statistical causal analysis. The future scope of the work includes dynamic vegetation model-driven hypothesis testing in the context of climate and LULC changes.

During the study period, we observed that LAI has increased significantly, which is consistent with the findings from previous studies^5,29^ . Previous literatures^5,29^ used MODIS LAI as their primary dataset. Chen et al.^5^ also used AVHRR LAI3g datasets along with MODIS LAI for consistency checking. They found both the datasets showed greening over India. The primary contributor to increasing LAI in India is agricultural expansion^5^. The annual time series of both forests and croplands show a steadily increasing trend. During this period, forest cover has increased by 0.25% (attributed to government initiatives regarding afforestation), while cropland has increased about 0.65%, the largest increase among LULC classes. This gives us an insight that the increase in LAI is mainly attributed to human management activity. However, we found a weak increase in MODIS GPP compared to an increase in MODIS LAI. FLUXCOM GPP changes are not significant over most of the regions. A global study by Zhang et al.^11^ also found a weak GPP increase compared to global greening from 2000 to 2015. Sarmah et al.^29^ found a mismatch between vegetation greening and GPP in South Asia. Sarmah et al. used both MODIS and PML-V2 GPP in their analysis. The outcome was consistent from these two different datasets. Our results agree with these findings. NPP has not shown any statistically significant changes across the country despite this greening. However, the trends of NPP have strong spatial variations, which are similar to those reported by Bejagam et al.^40^. Notably, the NPP from temporally consistent Indian forests shows a significant declining trend of 0.75 ± 0.58 TgC year^-2^ and has declined by 6.19% over the 19 years in the twenty-first century. The most prominent vegetation productivity decline has been witnessed in some of India's most biodiverse and pristine forest regions such as Northeast India and Western Ghats. We investigate further the key factors influencing the spatial variability in the NPP trend pattern. We discovered that regions with significant decreasing NPP trends are also associated with a significant warming pattern. These regions are also found to be the warming hotspots in the country. We concluded from different statistical methods that, decreases in NPP over these regions are attributed to warming. Decreasing photosynthesis and stable respiration, above a threshold temperature, over these regions, are the key reasons behind the declining NPP. The details underlying mechanism through which temperature impacts NPP have been discussed earlier in this section. Recent studies also show decreased crop yield due to warming^60^. This finding is probably one of the proxies that confirm the warming-induced declining vegetation productivity in India and agrees with our conclusions from the present analysis.

In the last two decades, the green cover of India has increased [MoEFCC, 2021; https://unfccc.int/sites/default/files/resource/INDIA_%20BUR-3_20.02.2021_High.pdf"] and Indian forests are still a net sink of carbon^61^; however, our study finds that this sink may be weakened due to warming.

Our analysis shows that climate change especially temperature rise has already started affecting vegetation productivity and carbon uptake in Indian forests. The study also conveys a strong scientific message that increasing greenness does not necessarily lead to increased carbon uptake, specifically for a country like India experiencing agricultural intensification. This analysis also has significant implications on the scientific analyses for planning to achieve net zero by 2070, as committed by India.

## Methods

### MODIS LAI product

Terra MODIS (MOD15A2H) Version 6 datasets are used to analyse vegetation greenness^7^. The spatial and temporal resolutions are 500 m and 8 days, respectively. The data quality control layer obtained from the data source is applied to remove the cloud-covered and bad pixels. In Fig. S25, we calculated the annual average percentage of good pixels taken from each grid. Annually, 82% to 89% were the good samples per grid, averaged across the grids, considered during the study period. We also presented a spatial map of overall good pixel count for annual scale at the grid level in Fig. S8a. We discovered that, on average, more than 85% of the pixels in each grid were taken from 2001 to 2019. Except for a very few grids, almost all the grids have more than 70% good pixels. Since good pixel count never reaches low (< 50%) for any grids, we have not performed any sensitivity analysis. We have also added good pixel count for non-monsoon (JFMAMOND) (Fig. S8b), and monsoon (JJAS) (Fig. S8c) periods. During the monsoon season (Fig. S8c) we observed a lower pixel count from some regions. However, during non-monsoon season (Fig. S8b) these Regions maintain a good pixel count. The data is processed as per the user guidelines^7^. We converted the 8-day data into Monthly data using a weighted average. There is a possibility that the first and last 8-day timesteps within a month have not contributed fully to that specific month. Hence, while converting 8-day data into a monthly scale, we had to assign weights to each timestep, depending on the number of days they are individually contributing to a month. The monthly averaged data then further converted into annual data by taking the mean value of each month.

### MODIS GPP and PSNnet product

In this study, we used MOD17A2HGF Version 6 Gross Primary Productivity (GPP) product and Net photosynthesis (PSNnet) product, with spatial and temporal resolutions are 500 m and 8 days respectively, for measuring vegetation productivity. MOD17A2HGF Version 6 achieved stage 3 validation^31^. This current version has not been validated globally, but previous versions of MOD17A2H GPP datasets have been validated globally across different biomes with flux tower GPP in many studies^62,63^ (R^2^ > 0.50). Net photosynthesis (PSNnet) is the difference between GPP and Leaf and Fine root Maintenance respiration (LFrMR). We primarily focused on the annual scale assessment of net vegetation productivity, which was carried out using the annual NPP data. However, for the explanation of the observed annual trend in NPP, we need to examine the net vegetation productivity at a finer temporal scale (monthly and 8 days). MODIS NPP datasets are only available on an annual scale. Thus, we have to switch to MODIS PSNnet dataset. We used the 8-day cumulative GPP and PSNnet products. These datasets also contain a data quality control layer which we applied for removing the cloud-covered and bad pixels. We have not performed any sensitivity analysis as mentioned earlier. We processed the data as per the user guidelines^31^ and converted these 8-day data sets of GPP and PSNnet into monthly and annual datasets using weighted average.

### MODIS NPP product

We obtained the Net Primary productivity from MOD17A3HGF Version 6 product; it gives us annual net primary productivity at 500-m spatial resolution Previous version of MOD17A3H dataset is consistent with flux tower NPP reflecting R^2^ value of 0.56^64^. Annual NPP is calculated by taking the difference between GPP and autotrophic respiration (RA) which is the sum of growth respiration (RG) and maintenance respiration (RM). This data is processed as per the user guidelines^65^.

### FLUXCOM GPP and NEE product

FLUXCOM GPP and NEE products are generated by 3 machine learning methods (RF, ANN, MARS) with the help of remote sensing data from MODIS (LST, NDVI, EVI, LAI, fPAR, BRDF) and meteorological data from CRUNCEPv6 (air temperature, precipitation, global radiation and VPD). The FLUEXCOM GPP and NEE datasets are available at Monthly scale at 0.5° × 0.5° resolution. The datasets are further evaluated with the eddy covariance data, for GPP R^2^ > 0.7 and for NEE R^2^ < 0.5^33,34^.

### MODIS LULC product

Changes in forest cover and croplands are calculated using MODIS MCD12Q1 datasets. This dataset gives us an annual land cover map at 500-m spatial resolution globally with different land cover legends. In this study, we used International Geosphere-Biosphere Programme classification to detect the land cover type. Evergreen needleleaf forests, evergreen broadleaf forests, deciduous needleleaf forests, deciduous broadleaf forests, and mixed forests are considered as the forest cover. For cropland, we considered the grids with 60% area as cultivated^66^.

### Precipitation and temperature data

Daily gridded precipitation data at 0.25° × 0.25° resolution provided by India Meteorological Department (IMD) is used in this study^67^. Daily average surface temperature is also obtained from IMD at 1° × 1° resolution^68^ . Monthly average and annual average precipitation and temperature are then obtained from these daily datasets. The precipitation and temperature data are taken for the period 2001–2019.

### Vapour pressure deficit (VPD) calculation

Vapour Pressure Deficit is the difference between saturation water vapour pressure and actual water vapour pressure. We calculated VPD by using Teten formula^69^ (Eq. 1).

1 $$ VPD = 611 \times e^{{\left( {\frac{17.27 \times t}{{t + 237.3}}} \right)}} \times \left( {1 - \left( {\frac{RH}{{100}}} \right)} \right) $$

Here t is the average temperature, RH is the relative humidity and the unit of VPD is Pa in Eq. 1. We used European Centre for Medium-Range Weather Forecasts (ECMWF) ERA5 monthly averaged reanalysis datasets of relative humidity and temperature at 0.25° × 0.25° spatial resolution at 1000 hPa level^70^ to calculate VPD. Monthly averaged VPD are then converted into annual averaged VPD.

### Photosynthetic active radiation (PAR) data

We have used CERES Photosynthetic Active Radiation (PAR) data to study the radiation changes. We have used Monthly mean CERES Surface PAR (Direct) data for all sky conditions. The Data has a spatial Resolution of 1° × 1°^71^.

### Calculation of trend

We used the modified Mann–Kendall test (taking into account the autocorrelation in time series)^72^ for detecting the trend. We use the significance level p ≤ 0.10 if not mentioned. After the detection of the trend, the slopes of the trend lines are obtained using linear regression. The percentage changes for 2001–2019 are calculated based on trend, duration, and initial value at 2001. They are computed by multiplying the annual statistically significant trend with the duration (18 years), divided by the initial value at the starting year, 2001.

### Kernel regression

Kernel regression, also known as kernel smoothing regression, approach is used to estimate the conditional expectation of a random variable (Y) given the regressors (X). Mathematically, it can be represented as E (Y|X) = f(X). Here, the response variable Y is GPP/LFfMR and X is Temperature. We have used the Nadaraya-Watson kernel regression model^73^ in our study. The general equation is given below.

$$ f\left( x \right) = \frac{{\mathop \sum \nolimits_{i = 1}^{n} K\left( {\frac{{x - x_{i} }}{h}} \right) \times y_{i} }}{{\mathop \sum \nolimits_{i = 1}^{n} K\left( {\frac{{x - x_{i} }}{h}} \right)}} $$

In the Equation, x_i_ observed value of the predictor, y_i_ is the observed value of the response variable, and x is the point where we want to estimate the expected value. The bandwidth parameter h determines the size of the kernel window around each data point. We have calculated the optimal bandwidth suggested by Bowman and Azzalini^74^. The number of points at which the kernel regression curve is estimated is defined by ‘n’. K is the Gaussian kernel function. f(x) is defined by the estimated conditional expectation of Y given X = x;

### Granger causality test

Granger causality test is a statistical hypothesis test that helps to find the cause-effect relationship between two time series. We say that 'X' is causing 'Y', only if we get a better prediction of 'Y' by including the past values of 'X' in the predictor^75^. In this study, we used the Granger causality test between the monthly datasets of Temperature (T), Precipitation (P), Vapour Pressure deficit (VPD), Photosynthetic Active Radiation (PAR), and Net photosynthesis (PSNnet)/Gross primary productivity (GPP) for finding the causal relationships among them. We detrended and deseasonalized each time series before applying the Granger Causality. We have considered a maximum lag of up to 11 months and significance level p ≤ 0.05. The detrended and deseasonalized time series are put into a vector autoregression (VAR) model and then "leave-one-out" type Granger causality test is performed. The null hypothesis is tested by conducting "chi-square test".

### Supplementary Information

Supplementary Information.

## Data Availability

No new data products are generated in this study. The datasets that are used in this study can be found in the following. Multiple MODIS products are used in this study: LAI (MOD15A2H), GPP (MOD17A2HGF), PSNnet (MOD17A2HGF), Annual NPP (MOD17A3HGF) and LULC (MCD12Q1). All the datasets can be downloaded from https://ladsweb.modaps.eosdis.nasa.gov**.** Monthly FLUXCOM GPP and NEE datasets at 0.5° × 0.5° spatial resolution can be downloaded from https://www.bgc-jena.mpg.de/geodb/projects/Data.php. India Meteorological Department (IMD) provided gridded daily rainfall data at 0.25° × 0.25° spatial resolution and gridded daily maximum temperature and minimum temperature at 1° × 1° spatial resolution. IMD gridded Rainfall and Temperature data can be downloaded from https://imdpune.gov.in/Clim_Pred_LRF_New/Grided_Data_Download.html. ECMWF reanalysis dataset of Monthly Relative Humidity and monthly mean temperature data at 0.25° × 0.25° spatial resolution are downloaded from https://cds.climate.copernicus.eu/cdsapp#!/dataset/reanalysis-era5-pressure-levels-monthly-means?tab=form to calculate VPD. Monthly mean CERES Surface PAR (Direct) data for all sky conditions at spatial Resolution of 1° × 1° can be downloaded from https://ceres.larc.nasa.gov/data/#synoptic-toa-and-surface-fluxes-and-clouds-syn.
