# Supplementary material for: Warming inhibits increases in vegetation net primary productivity despite greening in India
Source: Sci Rep. 2023 Dec 3;13:21309. doi: 10.1038/s41598-023-48614-3 (PMC10693629; doi:10.1038/s41598-023-48614-3)
Supplement: Supplementary file 1 — Supplementary Information. [file 41598_2023_48614_MOESM1_ESM.docx]

Supplementary Materials for

**Warming inhibits Increases in Vegetation Net Primary Productivity despite Greening in India**

Ripan Das, Rajiv Kumar Chaturvedi, Adrija Roy, Subhankar Karmakar, Subimal Ghosh*

*Corresponding author. Email:[subimal@civil.iitb.ac.in](about:blank)

**This Word file includes:**

Figures S1 to S25

Supplementary Texts 1 to 2

Supplementary Tables 1 to 2

Supplementary Figures:


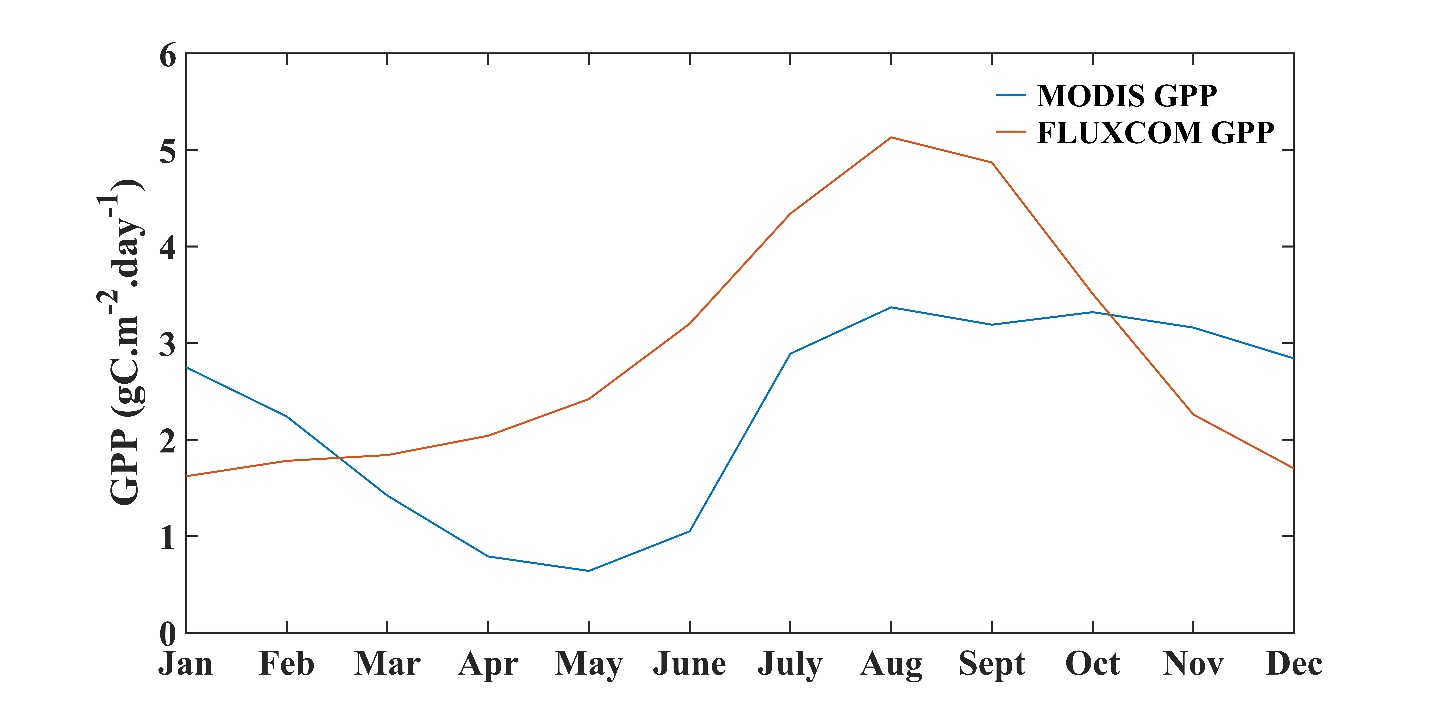


*Figure S1. Climatology of Vegetation Productivity: The climatology plot of MODIS GPP and FLUXCOM GPP over India during the period 2001-2018. Figure S1 is generated using MATLAB 2023a software (https://www.mathworks.com/?s_tid=mlh_gn_logo).*


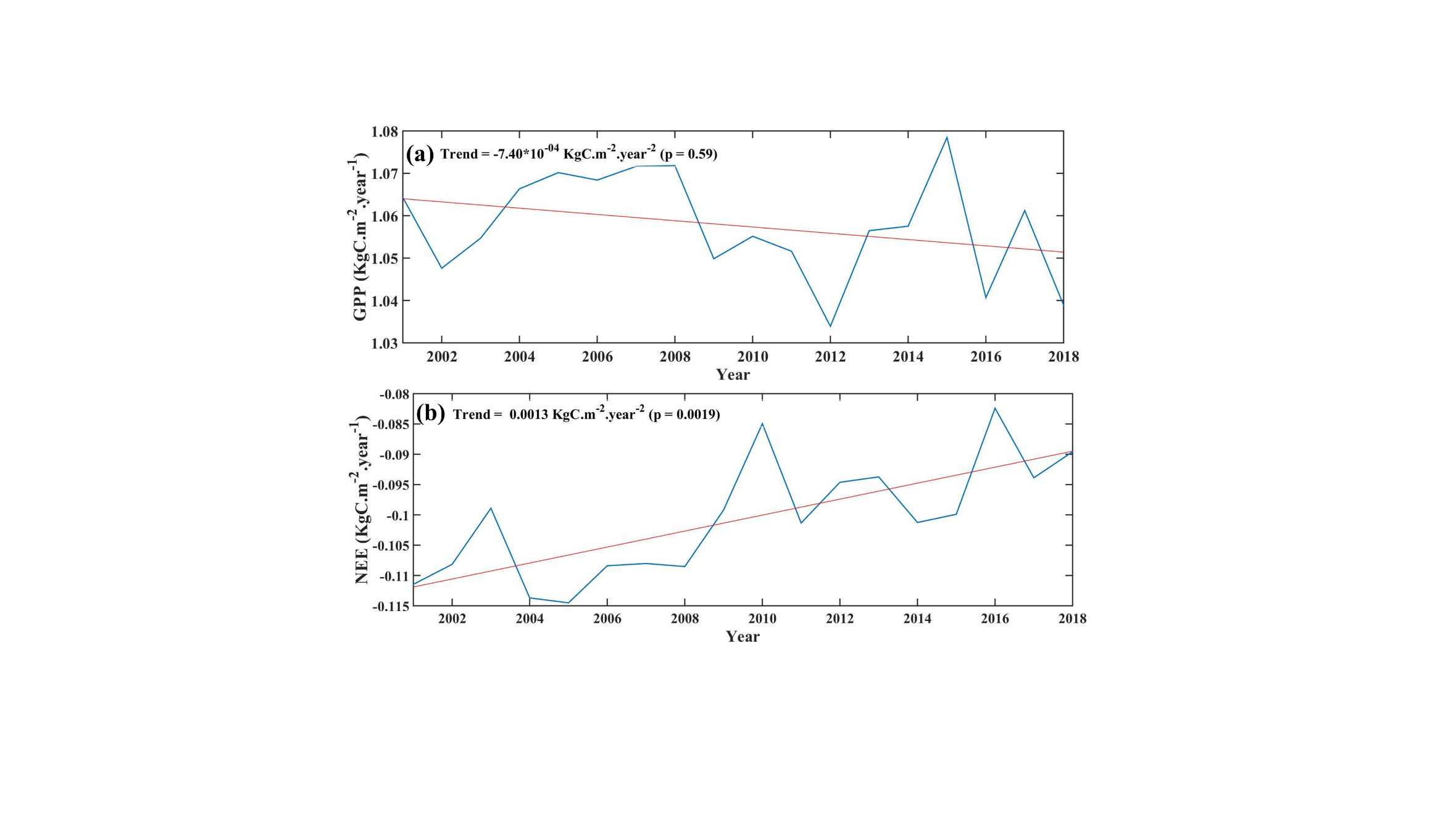


*Figure S2. Time series FLUXCOM datasets: The time series FLUXCOM GPP (a) and FLUXCOM NEE (b) over India during the period 2001-2018. Figure S2 is generated using MATLAB 2023a software (https://www.mathworks.com/?s_tid=mlh_gn_logo).*


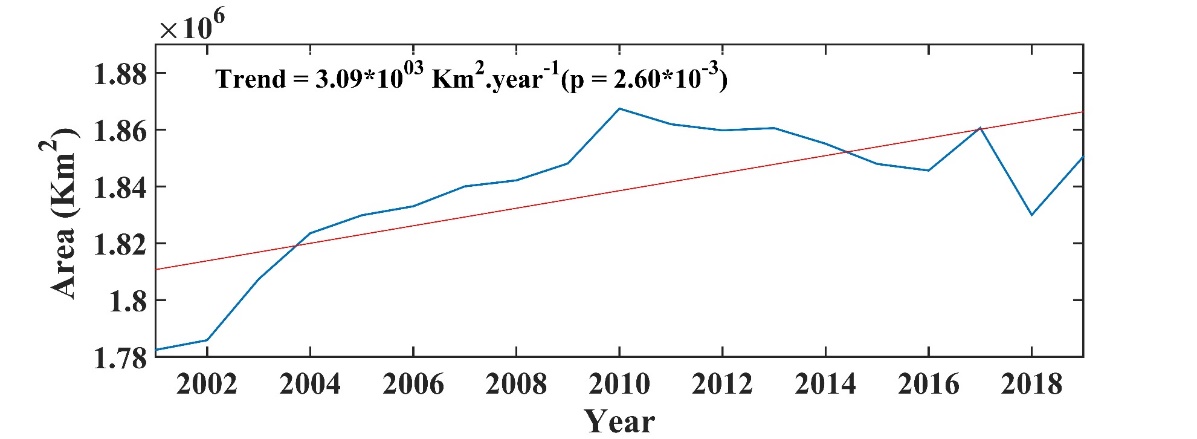

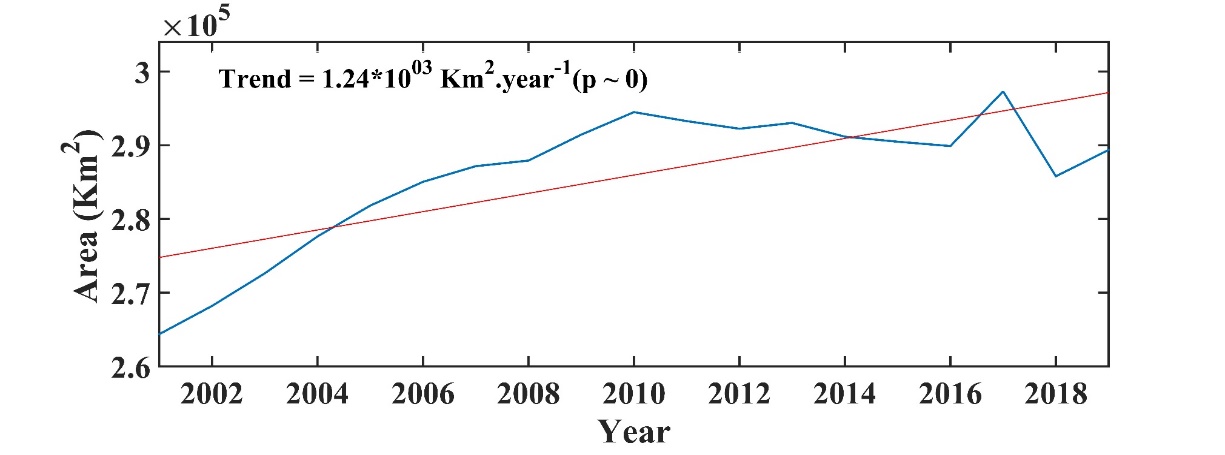


**(a)**

**(b)**

*Figure S3. Greening in India: The time series of forest cover area (a) and cropland area (b) over India during the period 2001 to 2019. Figure S3 is generated using MATLAB 2023a software (https://www.mathworks.com/?s_tid=mlh_gn_logo).*

**
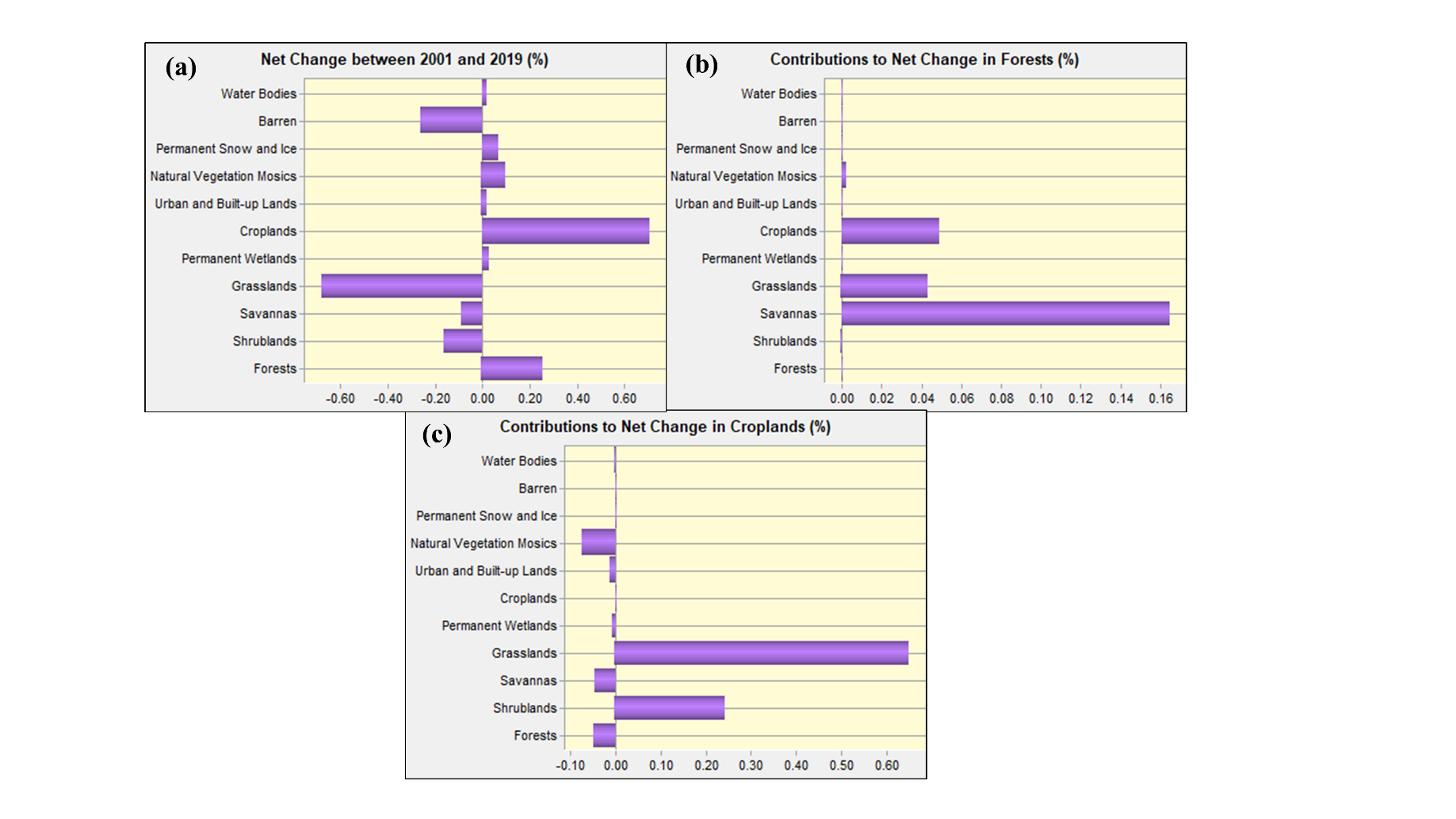
**

***Figure S4.*** ***Changes in area under LULC classes****:* *Net percentage changes in area under different LULC classes between 2001 and 2019 (a), contributions to net changes in forest area (%) (b), and contributions to net changes in cropland area (%) (c). Figure S4 is generated using Terrasat software in the Land Change Module (LCM) (https://clarklabs.org/).*


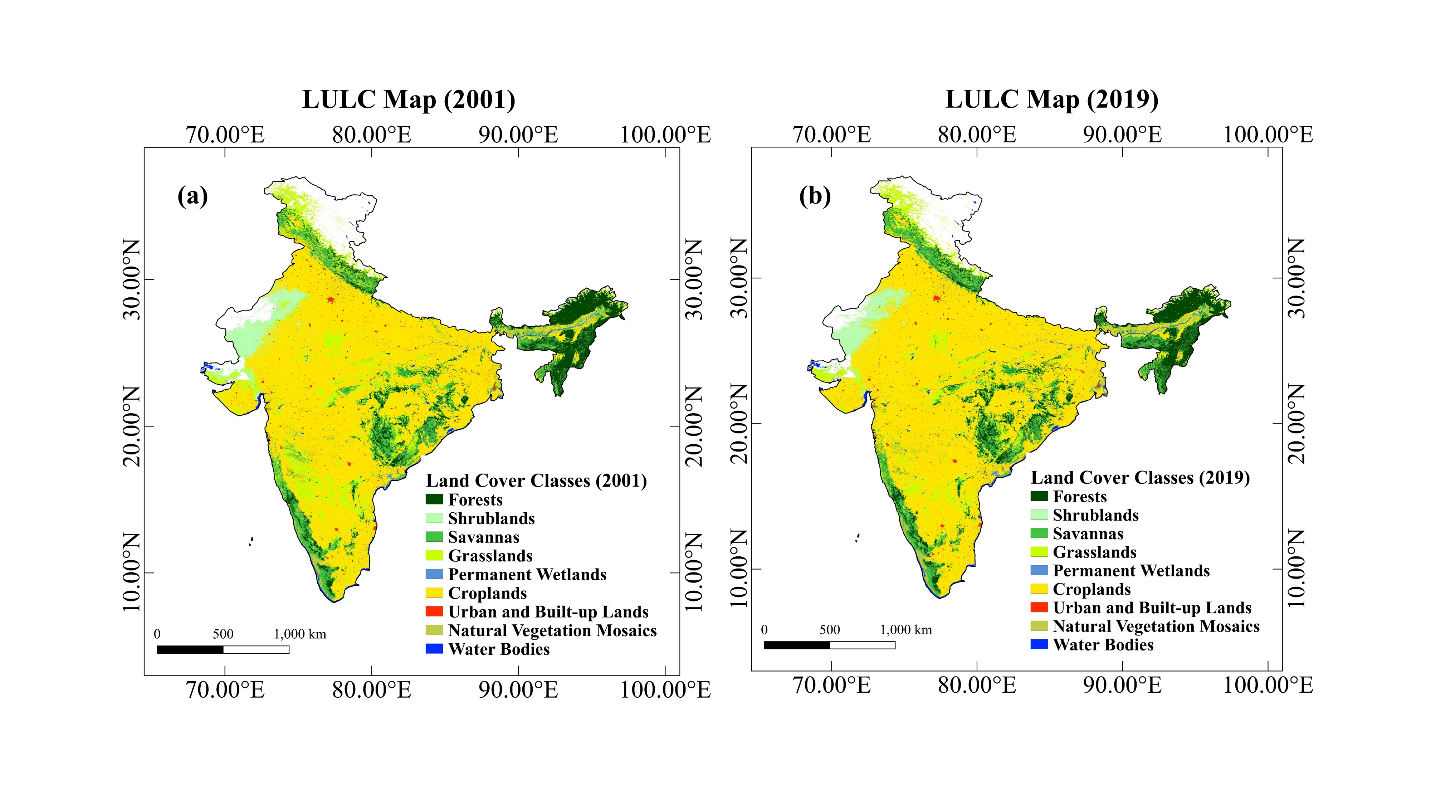


***Figure S5. LULC Map:*** *LULC Map of India for the years 2001 (a) and 2019 (b). Figure S5 is generated using QGIS 3.16 software (*[*https://www.qgis.org/en/site/forusers/download.html*](https://www.qgis.org/en/site/forusers/download.html)*).*


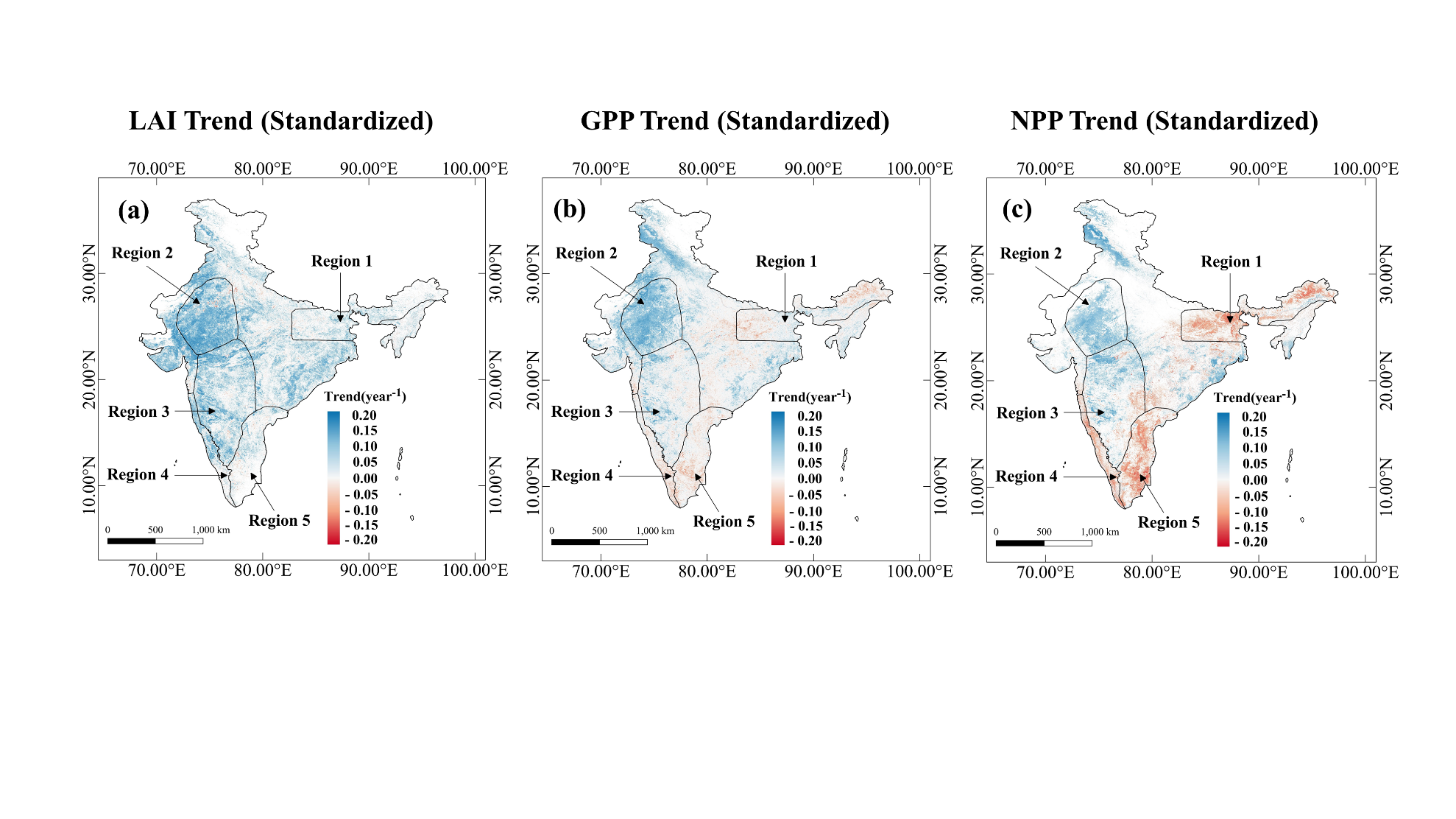


*Figure S6. Regional Trends of Standardized MODIS datasets: Trends of MODIS LAI (Standardized) (a), MODIS GPP (Standardized) (b), and MODIS NPP (Standardized) (c) during the period 2001-2019 at statistically significant level 0.1. Region 1: Northeast, Region 2: Northwest Arid, Region 3: Central Peninsular, Region 4: The Western Ghats, Region 5: East Coast Peninsular. Figure S6 is generated using QGIS 3.16 software (*[*https://www.qgis.org/en/site/forusers/download.html*](https://www.qgis.org/en/site/forusers/download.html)*).*


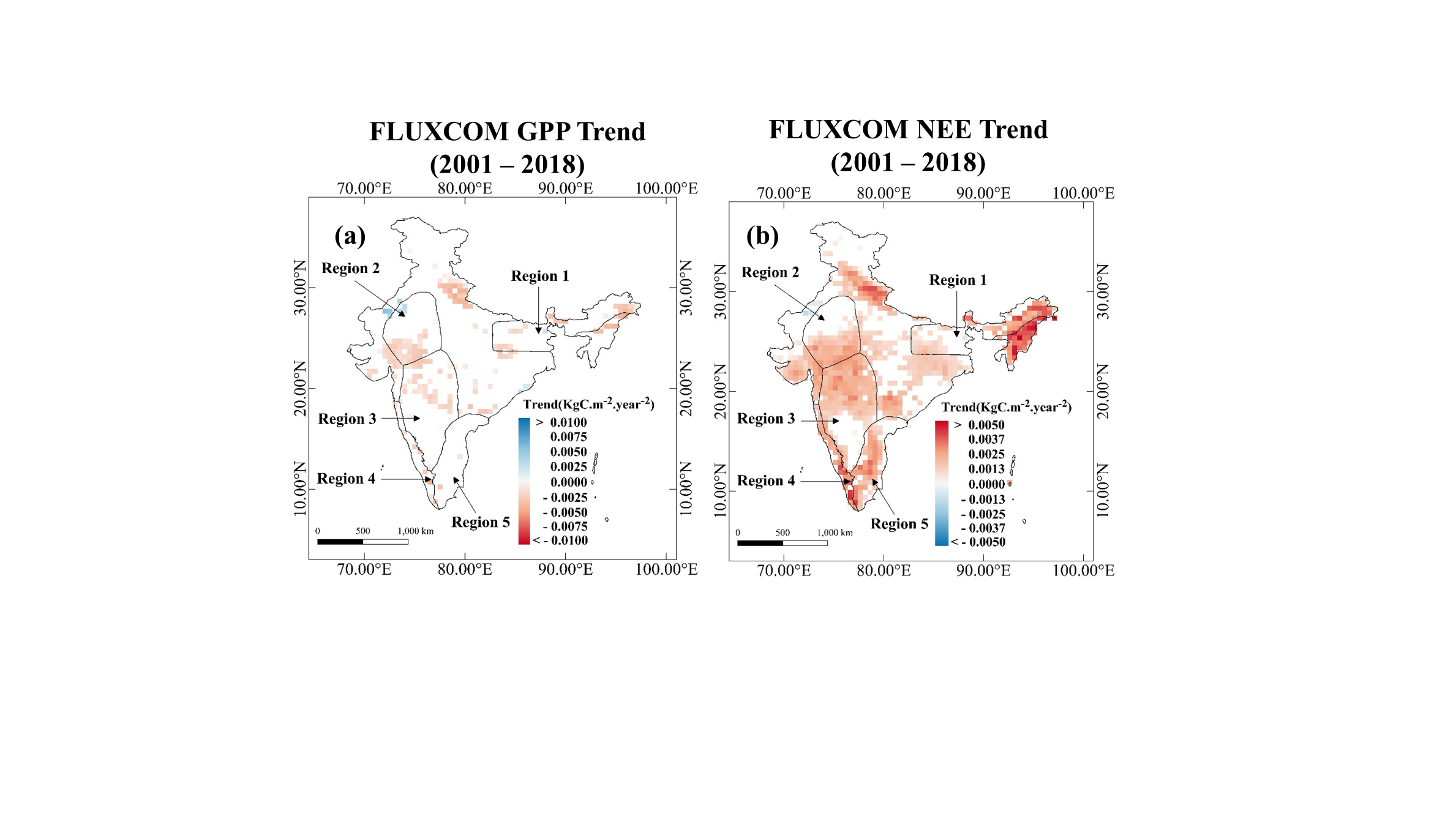


*Figure S7. Regional Trends of FLUXCOM datasets: Trends FLUXCOM GPP (a), and FLUXCOM NEE (b) during the period 2001-2018 at statistically significant level 0.1, Region 1: Northeast, Region 2: Northwest Arid, Region 3: Central Peninsular, Region 4: The Western Ghats, Region 5: East Coast Peninsular. Figure S7 is generated using QGIS 3.16 software (*[*https://www.qgis.org/en/site/forusers/download.html*](https://www.qgis.org/en/site/forusers/download.html)*).*


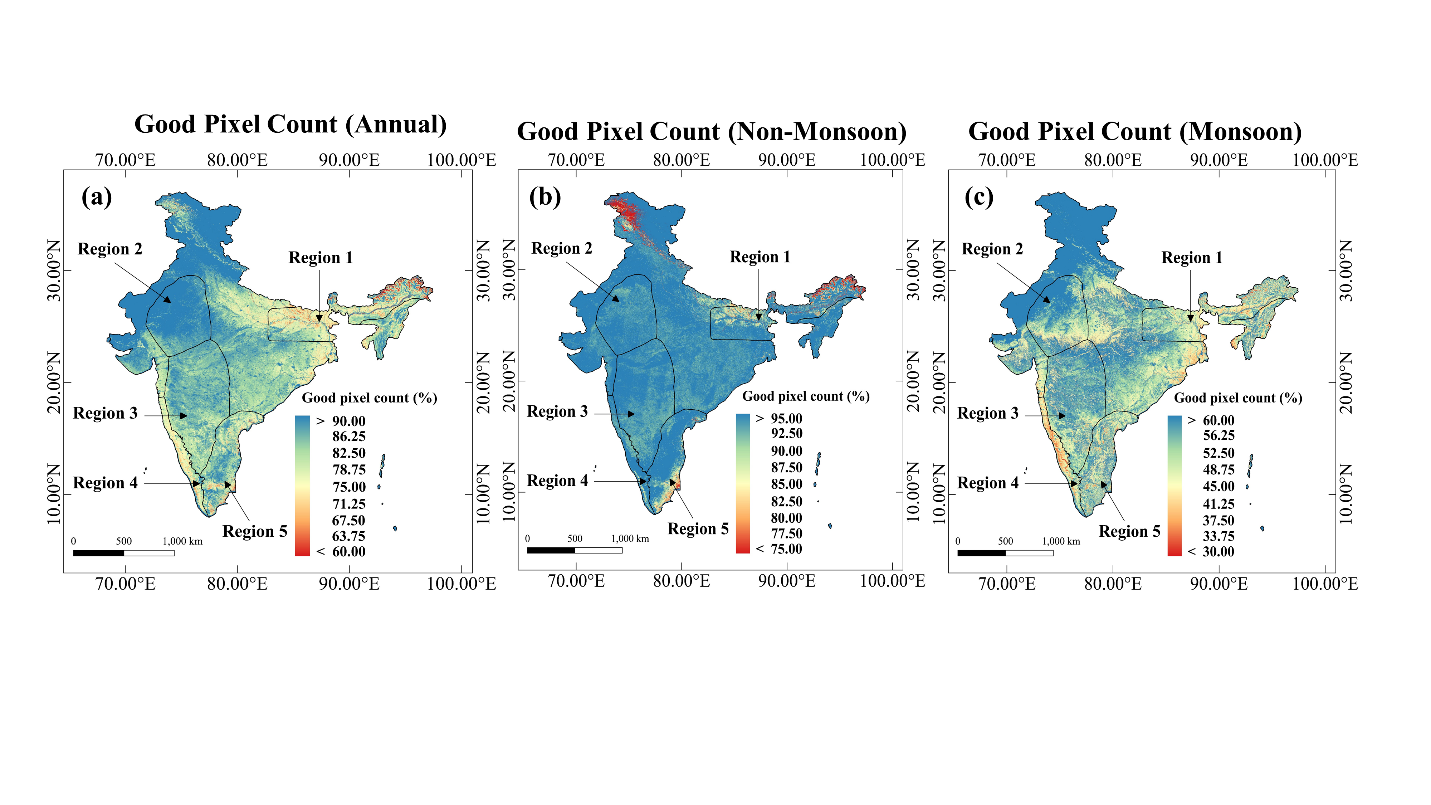


***Figure S8. Satisfactory Pixel count****: Spatial map of good pixel count (percentage) for annual (a), non-monsoon (b) and monsoon (c) over India during the period2001-2019. Figure S8 is generated using QGIS 3.16 software (*[*https://www.qgis.org/en/site/forusers/download.html*](https://www.qgis.org/en/site/forusers/download.html)*).*


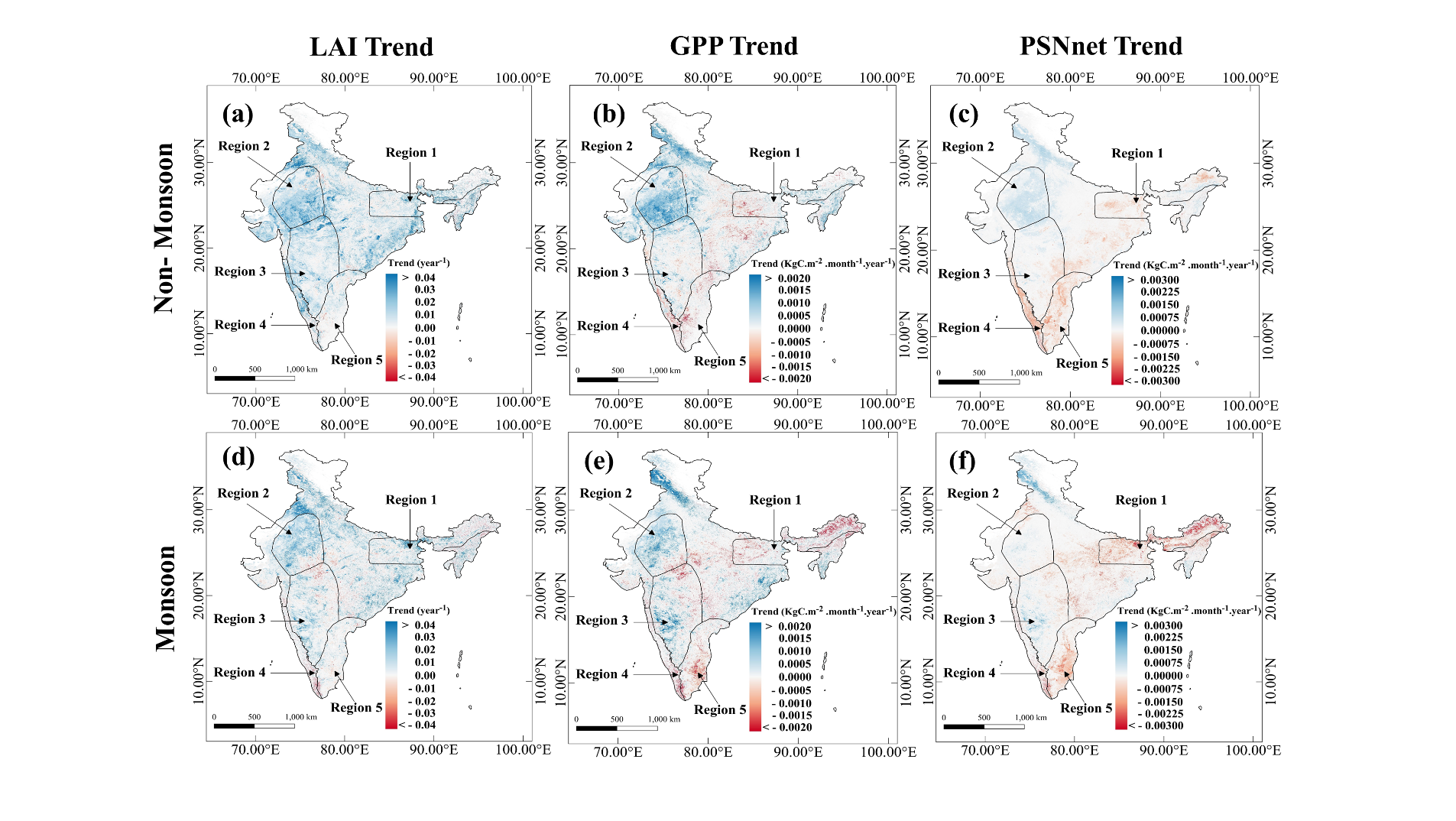


***Figure S9. Seasonal Trends*:** *Trends of LAI (a,d), GPP (b,e), and PSNnet (c,f) at statistically significant level 0.1 during non-monsoon season (a,b,c) and monsoon season (d,e,f) during the period 2001-2019. Region 1: Northeast, Region 2: Northwest Arid, Region 3: Central Peninsular, Region 4: The Western Ghats, Region 5: East Coast Peninsular. Figure S9 is generated using QGIS 3.16 software (*[*https://www.qgis.org/en/site/forusers/download.html*](https://www.qgis.org/en/site/forusers/download.html)*).*

***
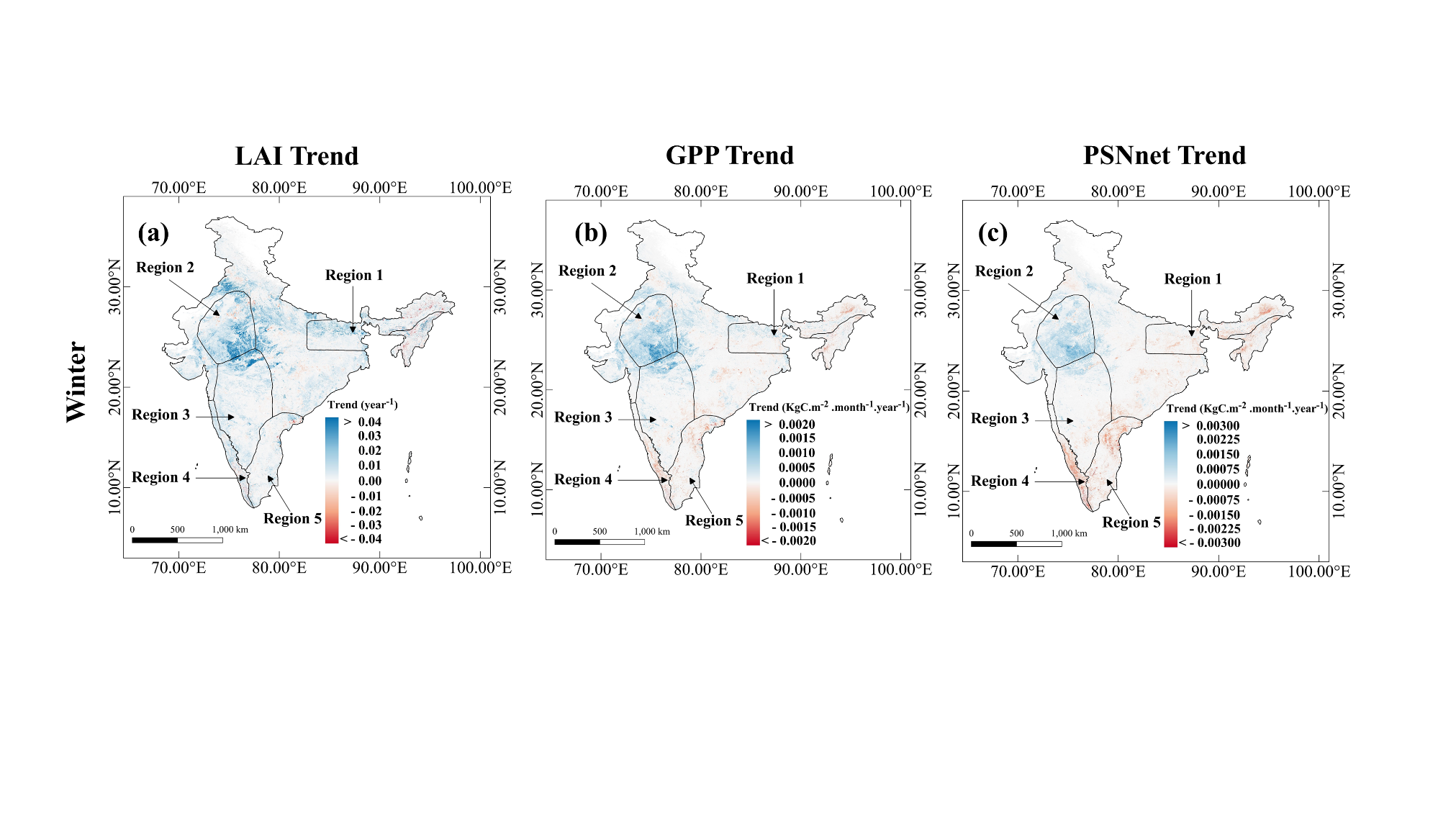
***

***Figure S10. Winter Trends*:** *Trends of LAI (a), GPP (b), and PSNnet (c) at statistically significant level 0.1 during winter (DJF) season 2001-2019. Region 1: Northeast, Region 2: Northwest Arid, Region 3: Central Peninsular, Region 4: The Western Ghats, Region 5: East Coast Peninsular. Figure S10 is generated using QGIS 3.16 software (*[*https://www.qgis.org/en/site/forusers/download.html*](https://www.qgis.org/en/site/forusers/download.html)*).*


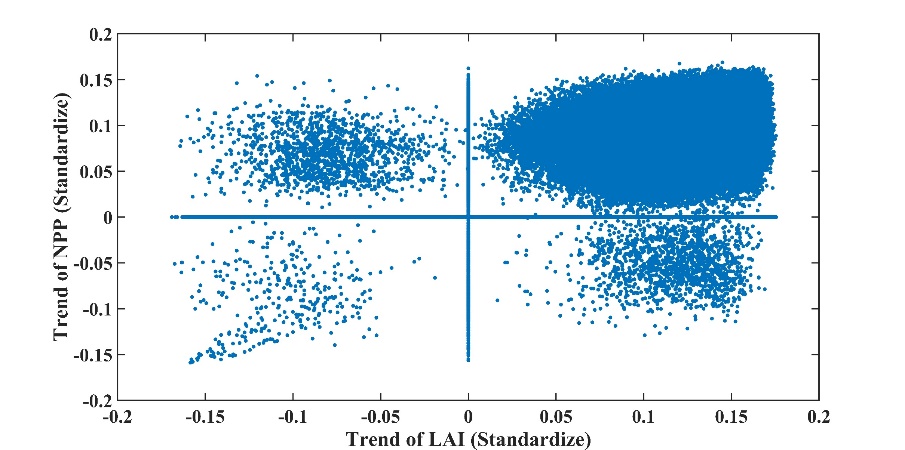

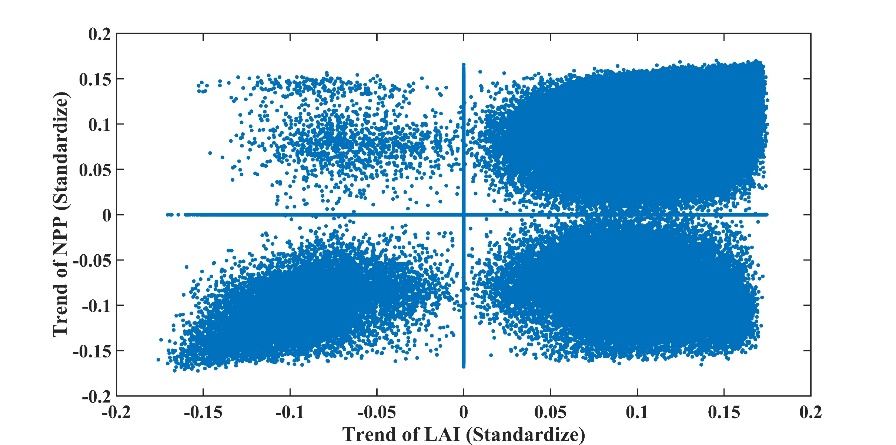

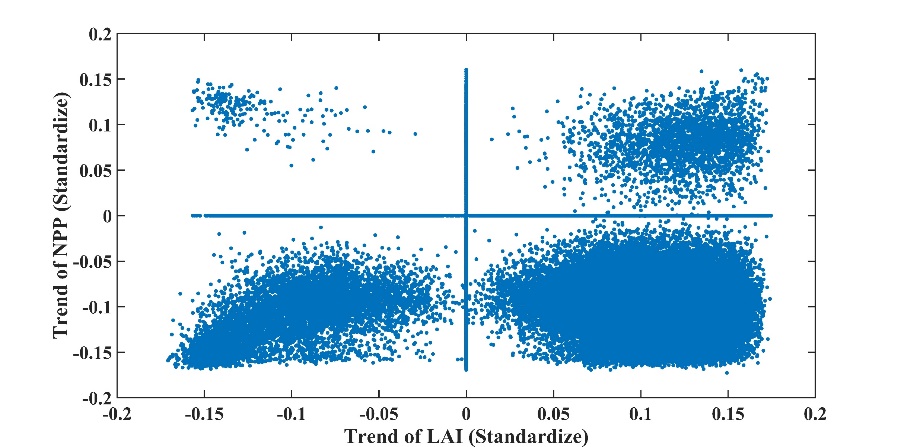

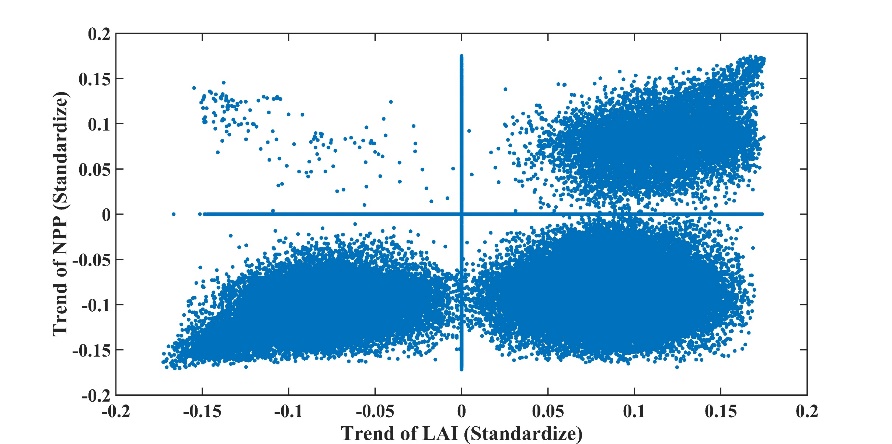

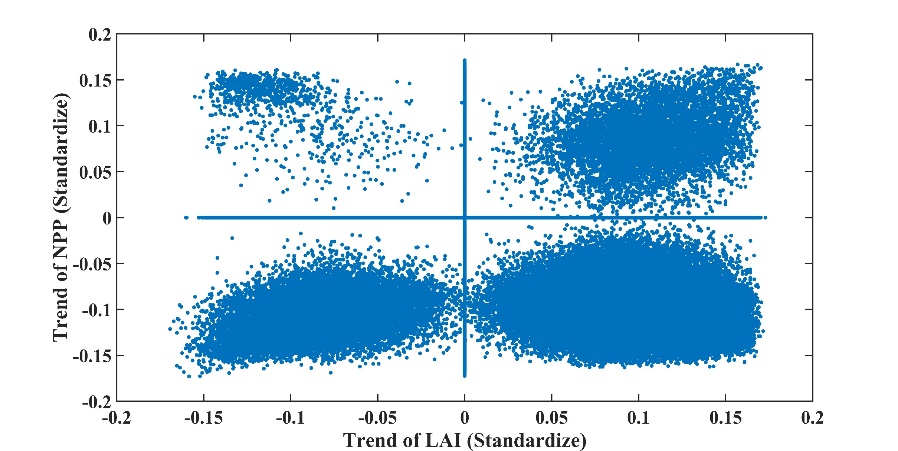


**(a)**

**(b)**

**(c)**

**(d)**

**(e)**

Supplementary Figure S7

*Figure S11. Divergence of Trend (Scatter): Scatter plot of trends of standardize LAI and NPP for Region 1: Northeast (a), Region 2: Northwest Arid (b), Region 3: Central Peninsular (c), Region 4: The Western Ghats (d) and Region 5: East Coast Peninsular (e) during 2001-2019. Figure S11 is generated using MATLAB 2023a software (https://www.mathworks.com/?s_tid=mlh_gn_logo).*


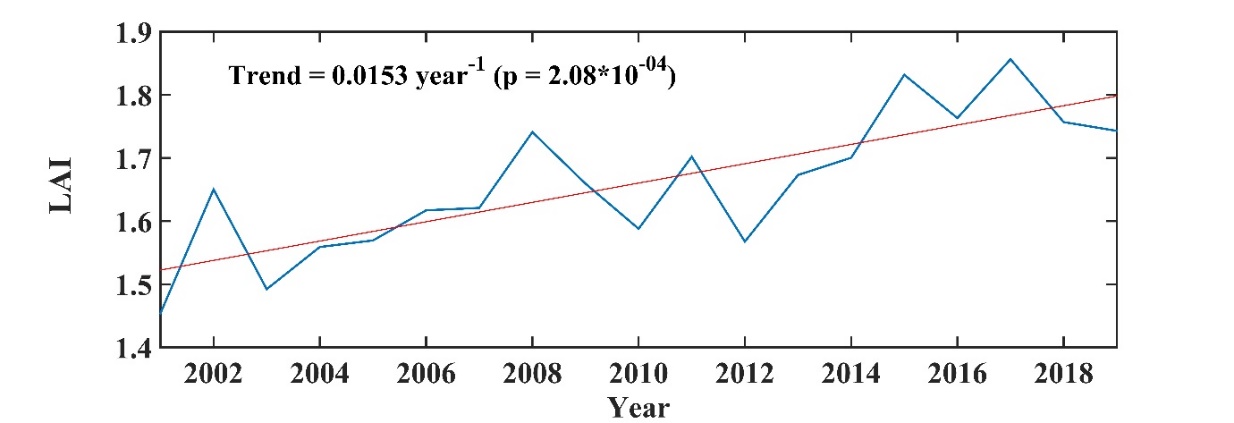

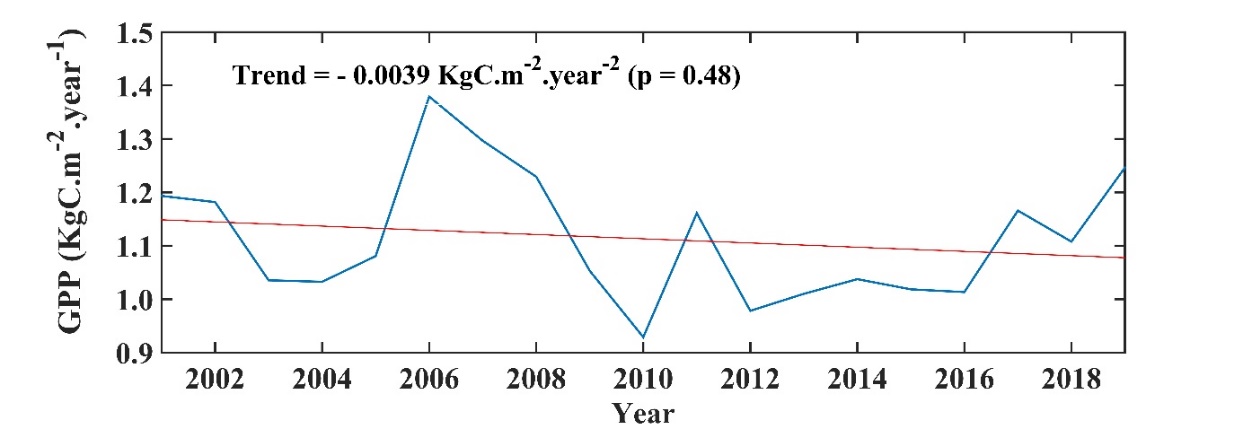


**(a)**

**(b)**


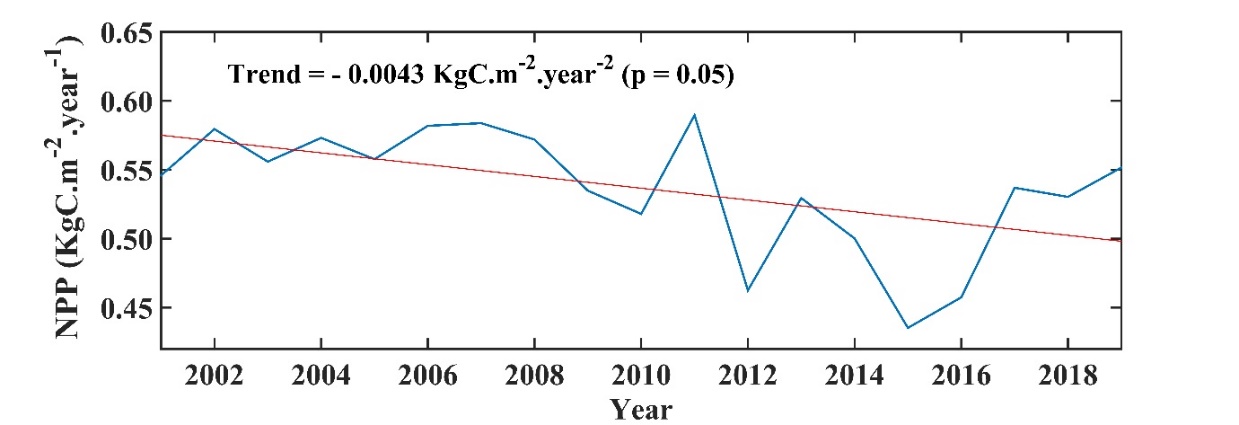


**(c)**

*Figure S12. Regional Trend in the Northeast India: The time series of LAI (a), GPP (b) and NPP (c) over Region 1: Northeast India during 2001-2019. Figure S12 is generated using MATLAB 2023a software (https://www.mathworks.com/?s_tid=mlh_gn_logo).*


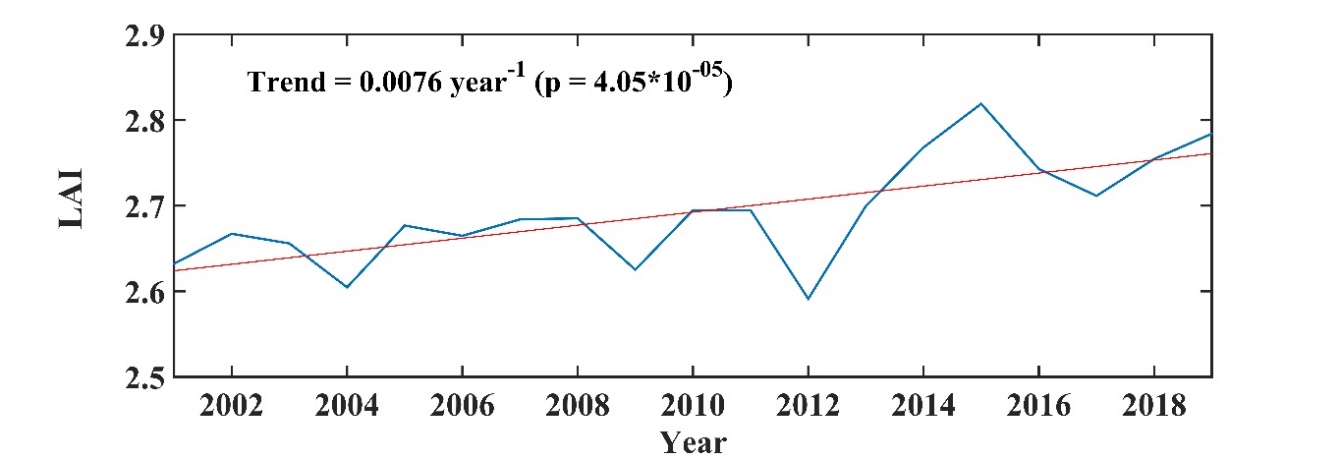

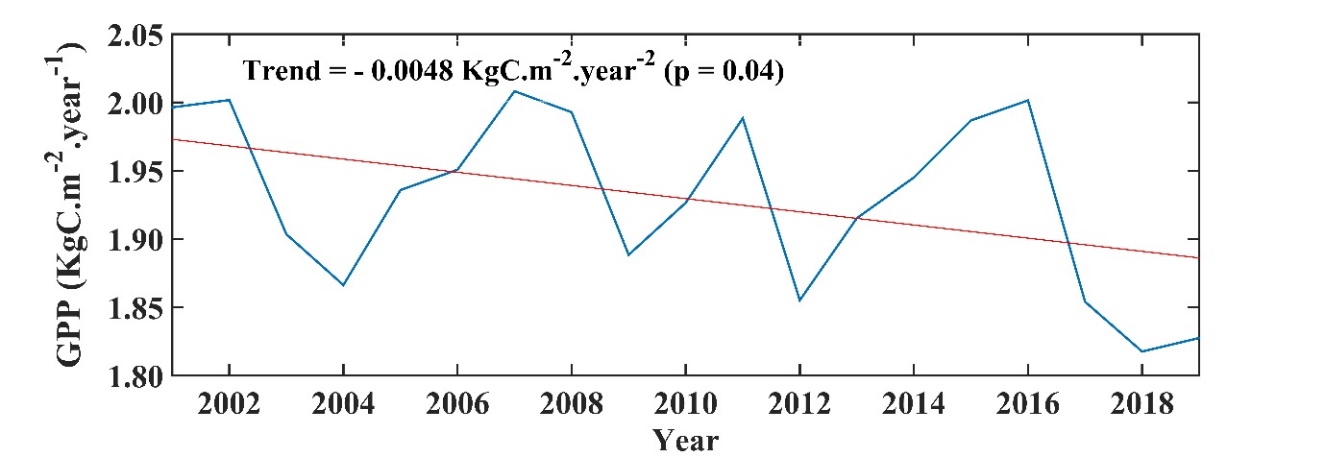

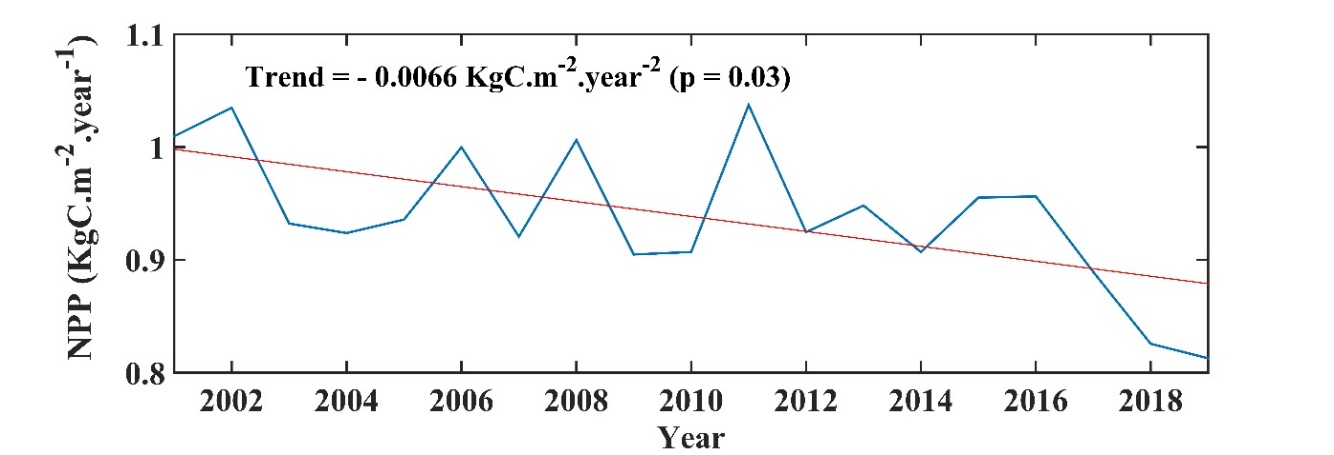


**(a)**

**(b)**

**(c)**

*Figure S13. Regional Trend in the Western Ghats: The time series of LAI (a), GPP (b) and NPP (c) over Region 4: The Western Ghats during 2001-2019. Figure S13 is generated using MATLAB 2023a software (https://www.mathworks.com/?s_tid=mlh_gn_logo).*


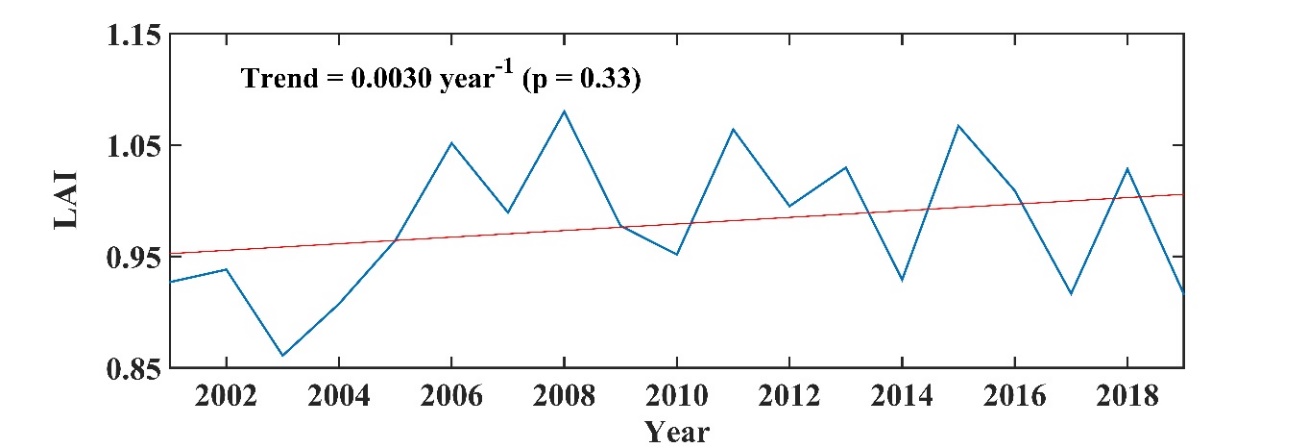

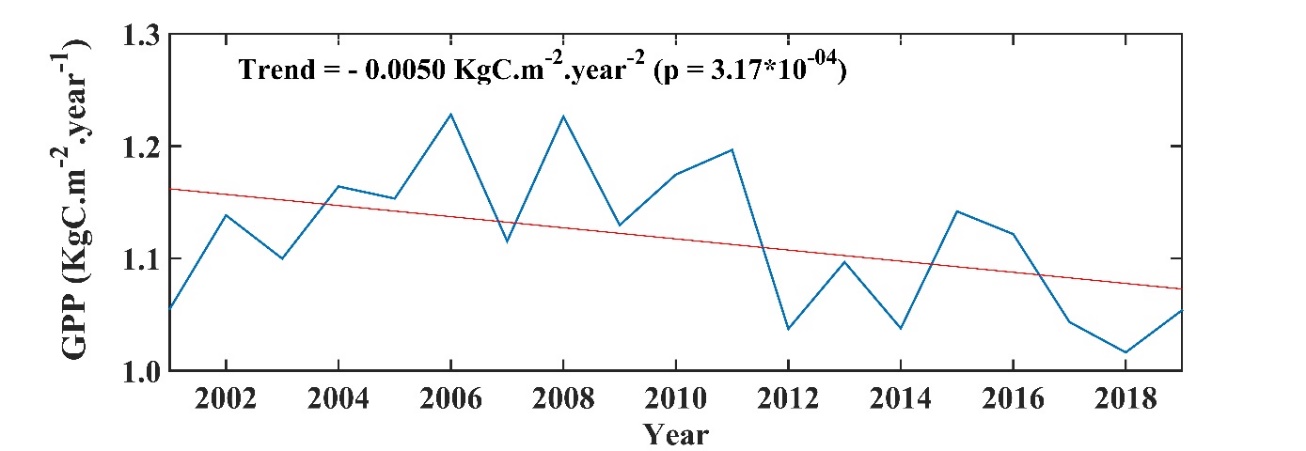

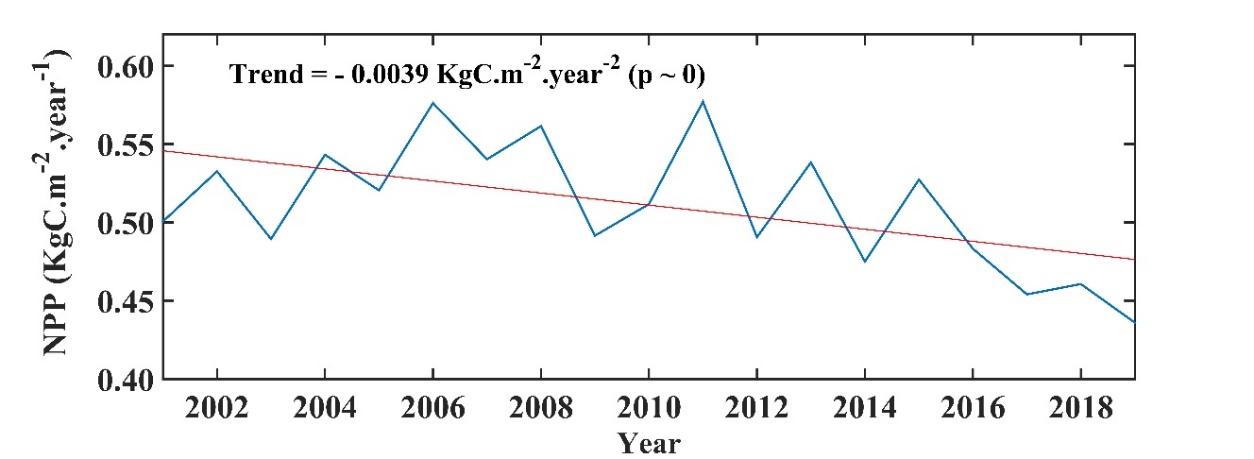


**(a)**

**(b)**

**(c)**

*Figure S14. Regional Trend in the East Coast Peninsular India: The time series of LAI (a), GPP (b) and NPP (c) over Region 5: East Coast Peninsular during 2001-2019. Figure S14 is generated using MATLAB 2023a software (https://www.mathworks.com/?s_tid=mlh_gn_logo).*

**
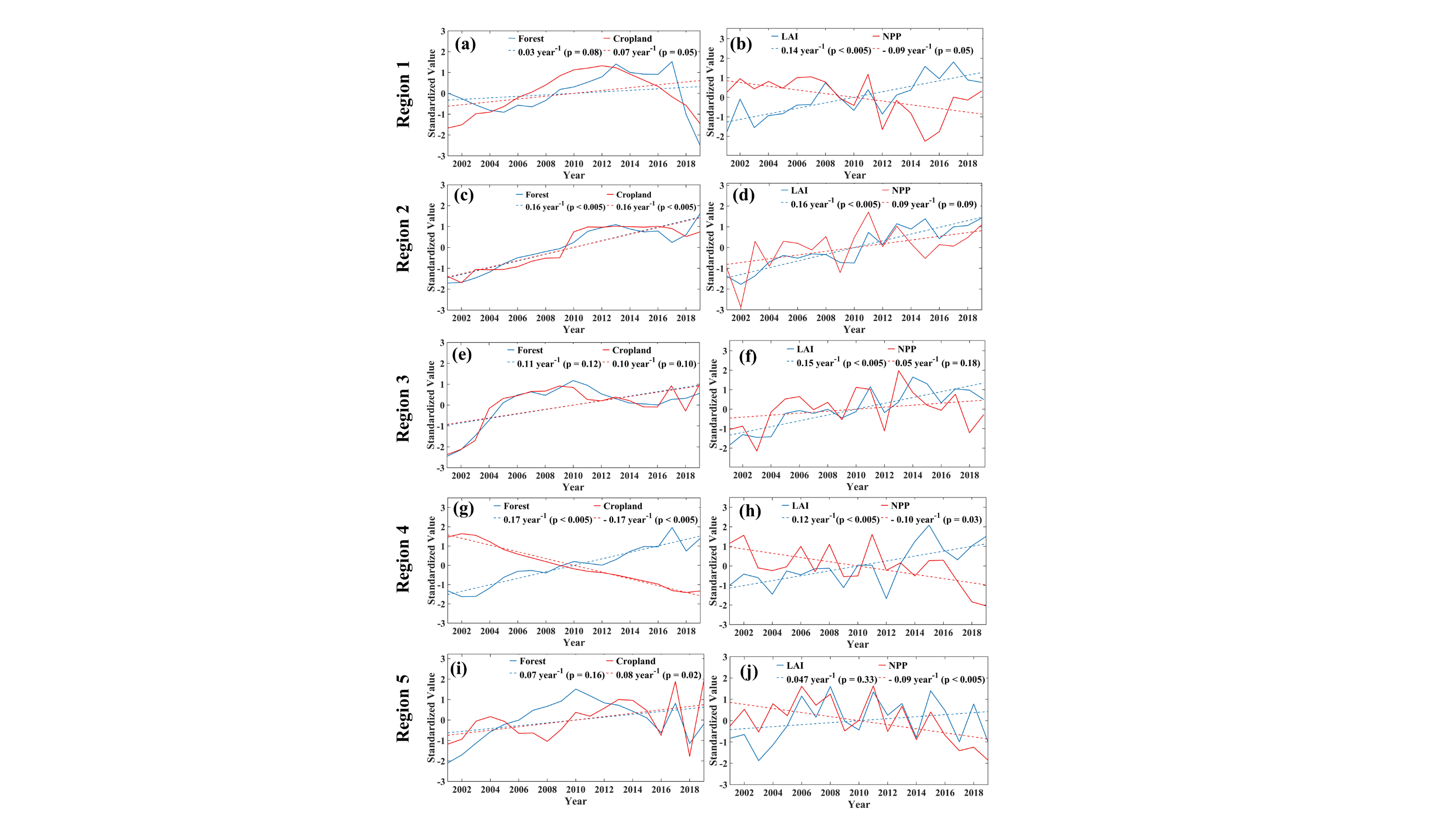
*Figure S15 Trends in cropland area, forest area, LAI and NPP:*** *Time series (2001-2019) of Standardized values of forest land area and cropland area in Region 1 (a), 2(c), 3 (e), 4 (g) and Region 5 (i). Time series of Standardized values of LAI and NPP in Region 1 (b), 2(d), 3 (f), 4 (h) and Region 5 (j). The dotted line represents the trends in corresponding variables. Figure S15 is generated using MATLAB 2023a software (https://www.mathworks.com/?s_tid=mlh_gn_logo).*


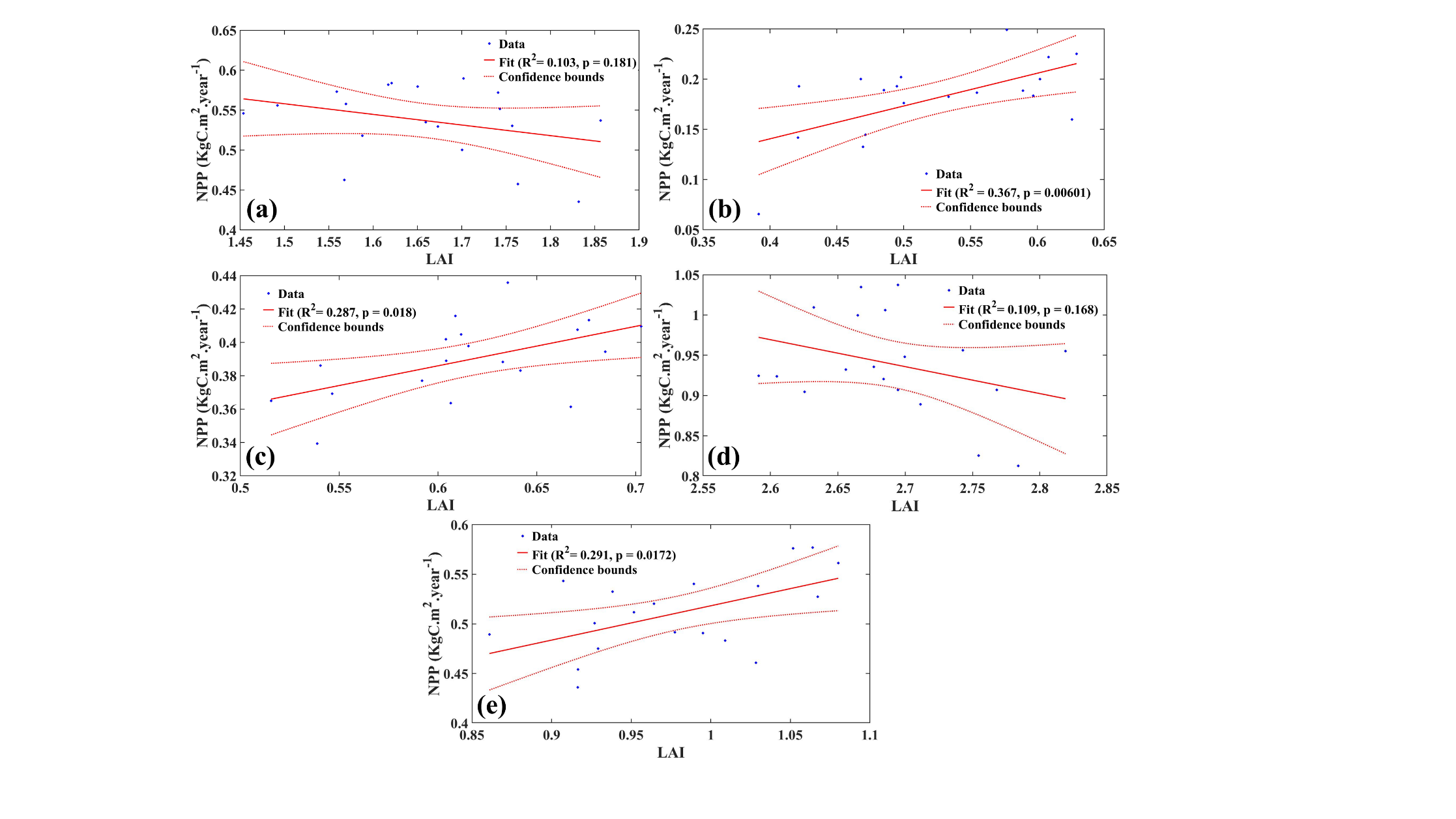


*Figure S16. Relationship between LAI and NPP at Regional scale: Liner Regression between annual LAI and NPP for Regions 1(a), 2(b), 3(c), 4(d), and 5 (e) during 2001-2019. Figure S16 is generated using MATLAB 2023a software (https://www.mathworks.com/?s_tid=mlh_gn_logo).*

**
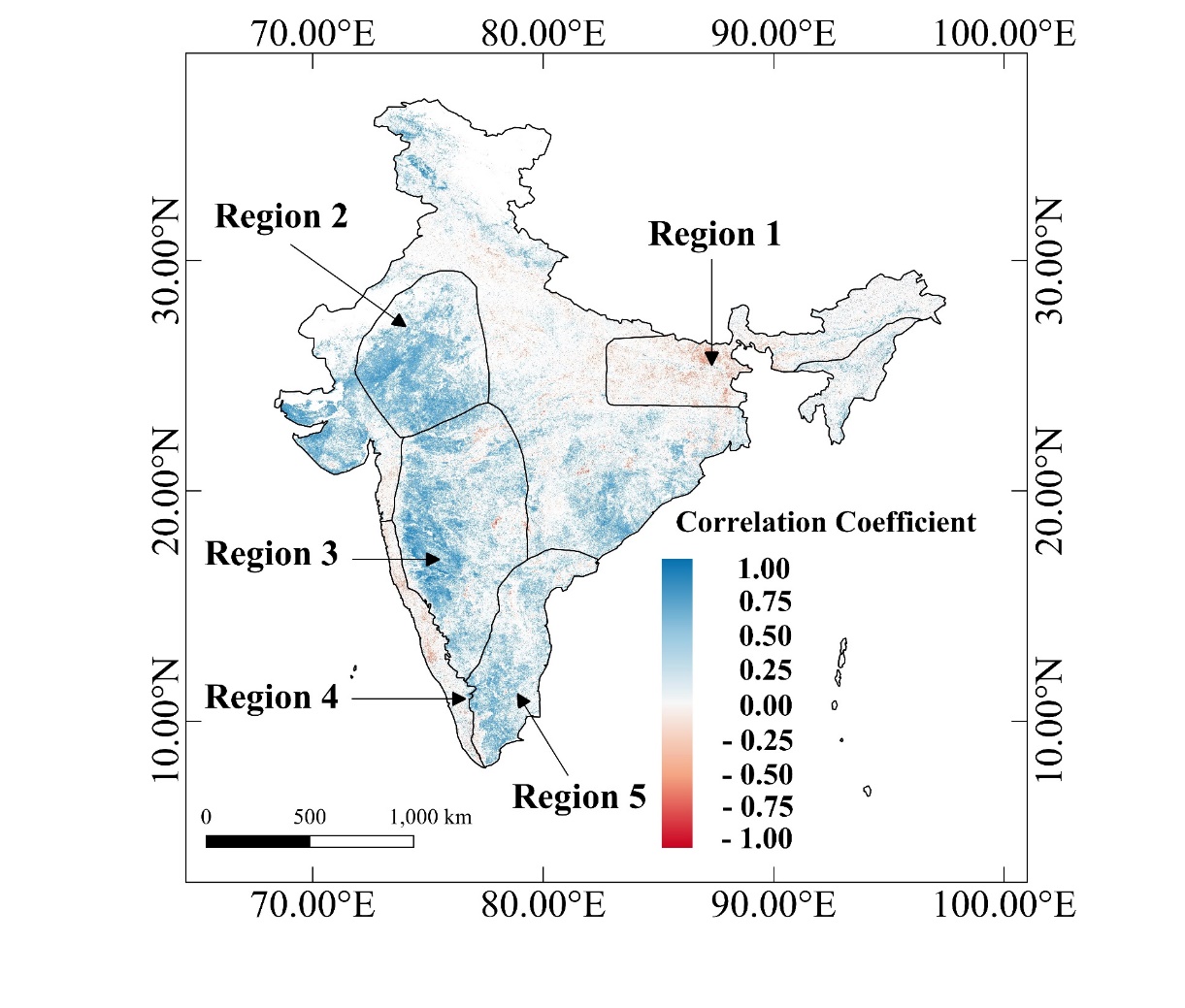
**

*Figure S17. Correlation between LAI and NPP: Pearson correlation coefficient map between NPP and annual average LAI during the period 2001-2019 at statistically significance level of 0.1. Figure S17 is generated using QGIS 3.16 software (*[*https://www.qgis.org/en/site/forusers/download.html*](https://www.qgis.org/en/site/forusers/download.html)*).*


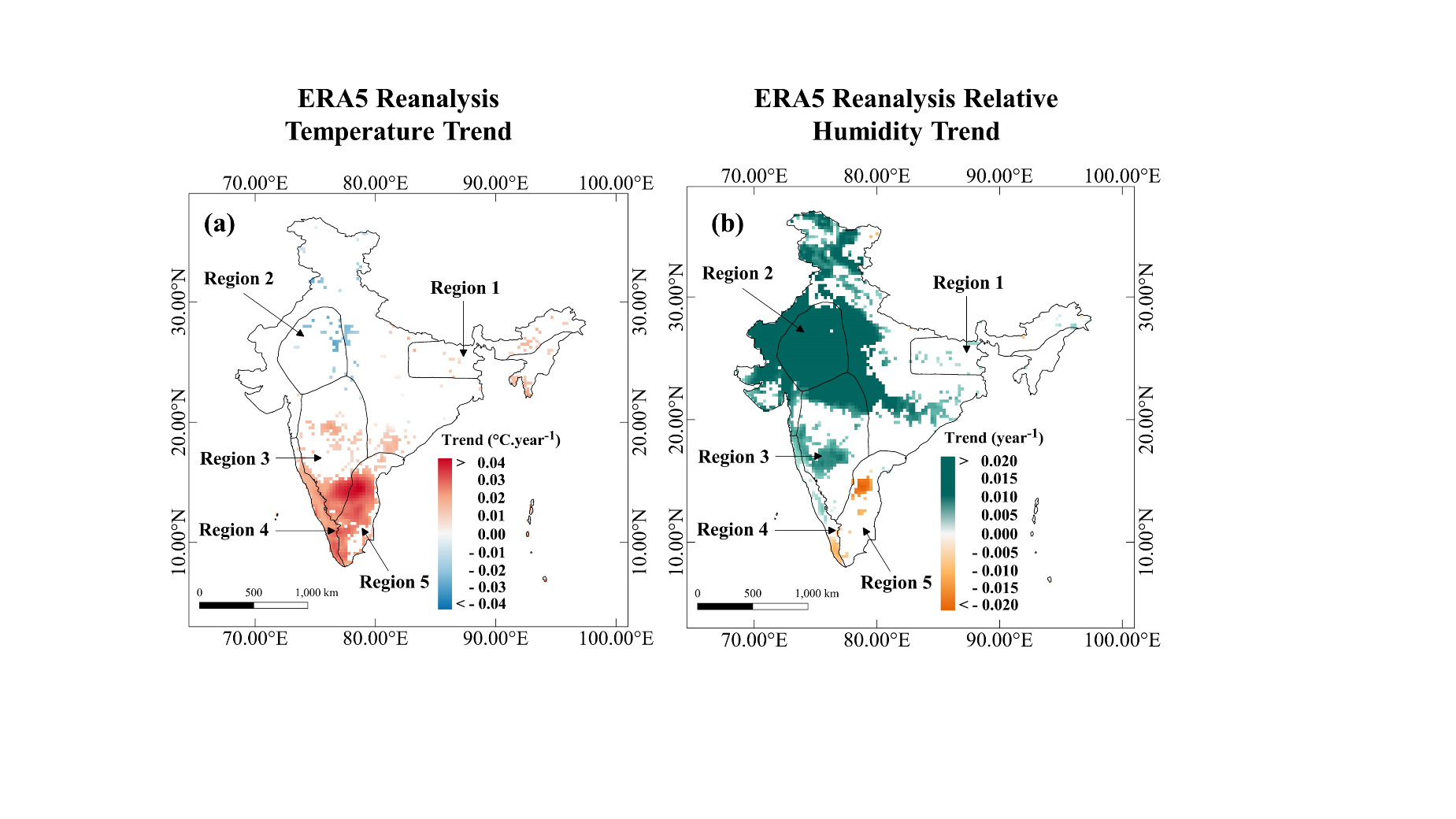


***Figure S18. Temperature Trends and Relative Humidity:*** *Trend of ECMWF Temperature (a) and ECMWF Relative Humidity (b) in India for the period 2001-2019. The figures only show statistically significant trends at 0.1 level. Figure S18 is generated using QGIS 3.16 software (*[*https://www.qgis.org/en/site/forusers/download.html*](https://www.qgis.org/en/site/forusers/download.html)*).*


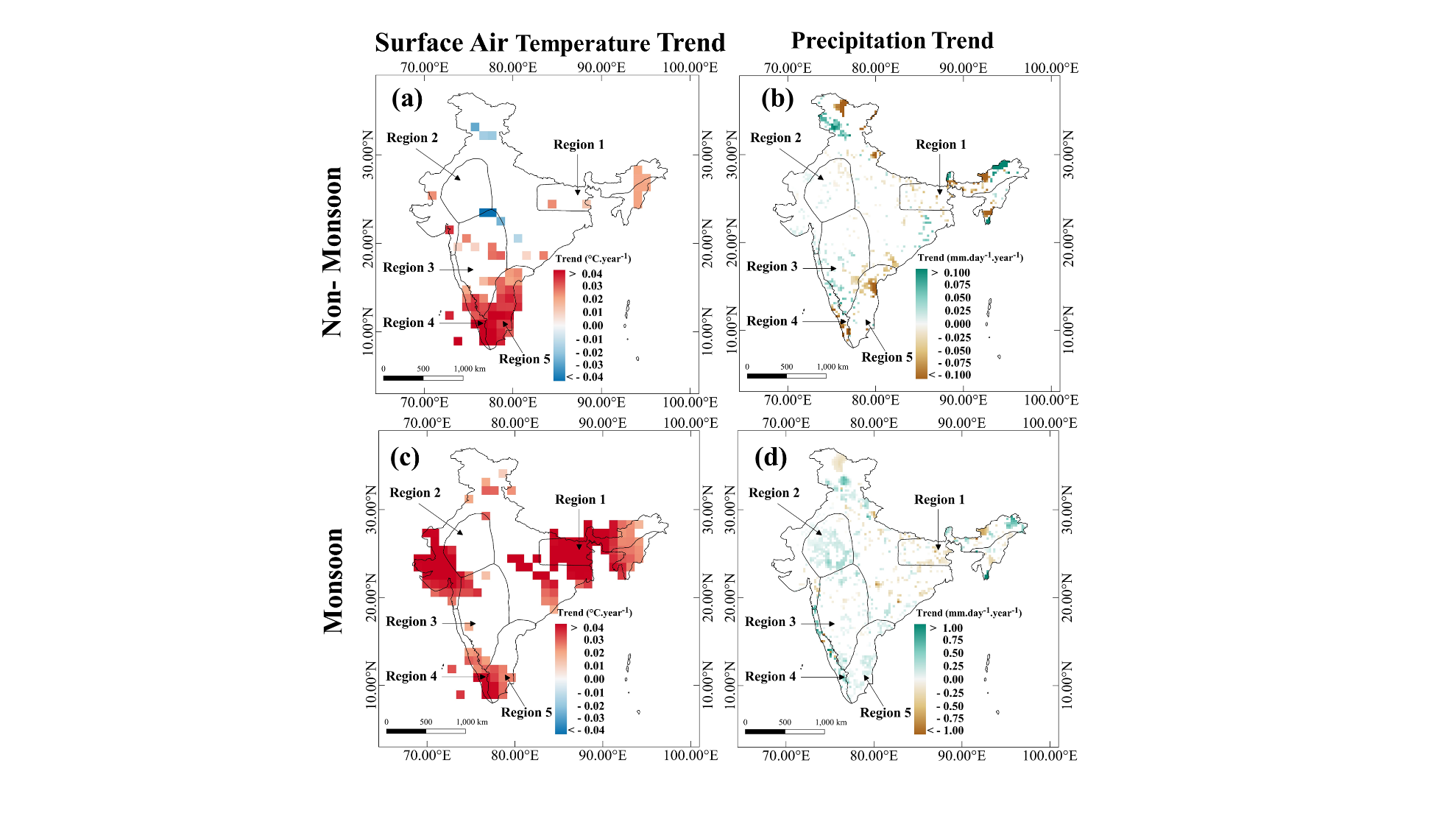


***Figure S19. Seasonal Trends in temperature and precipitation:*** *Trends of Surface Air Temperature (a,c) and Precipitation (b,d ) at a statistically significant level of 0.1 during the non-Monsoon period(a,b,) and Monsoon period (d,e) during 2001-2019. Figure S19 is generated using QGIS 3.16 software (*[*https://www.qgis.org/en/site/forusers/download.html*](https://www.qgis.org/en/site/forusers/download.html)*).*


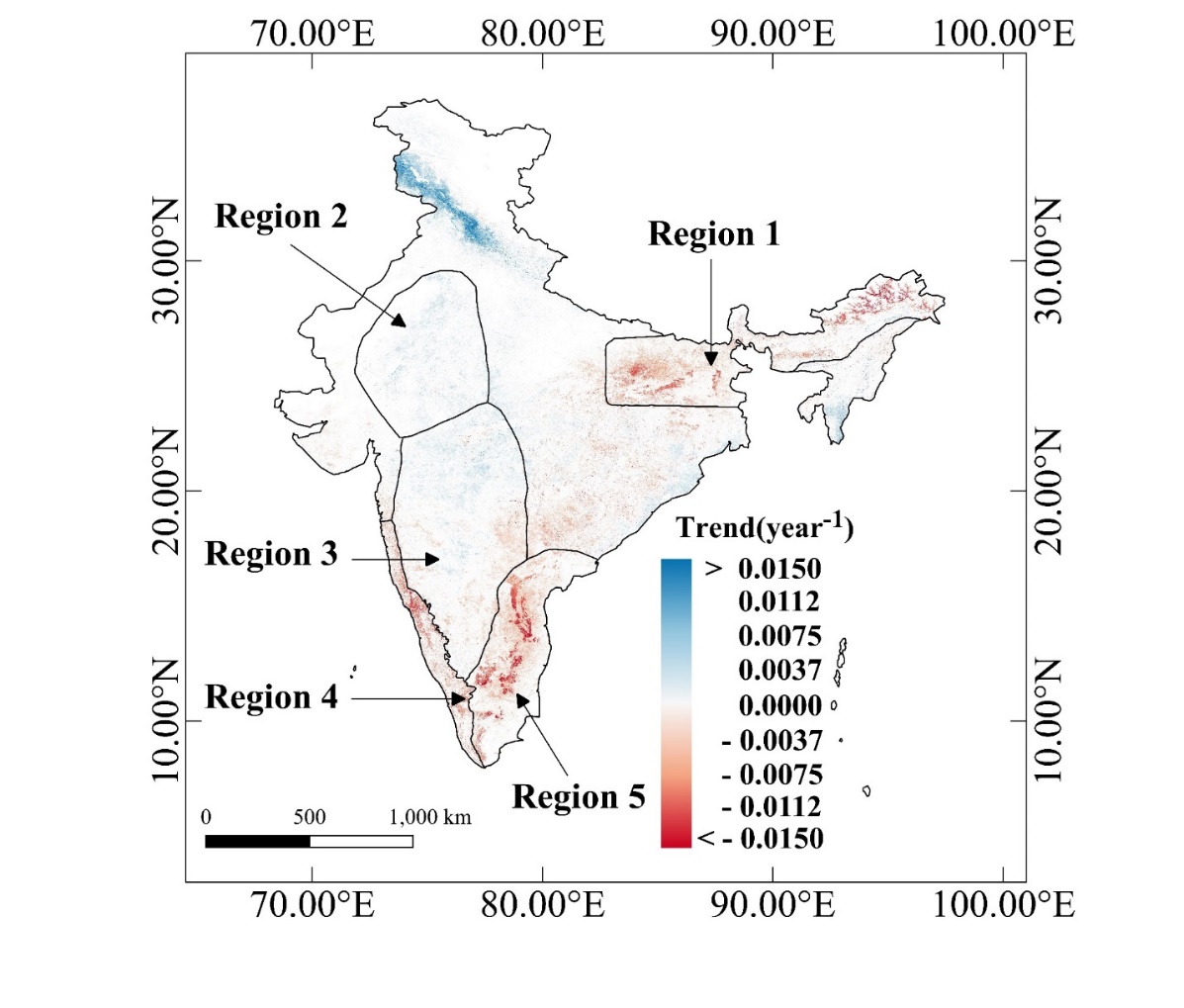


***Figure S20. Residual Trend Map:*** *We linearly regressed NPP as function of LAI (NPP = f (LAI)) at each pixel during the period 2001-2019 over India and calculated the trend of the Residuals at a statistically significance level of 0.1. Figure S20 is generated using QGIS 3.16 software (*[*https://www.qgis.org/en/site/forusers/download.html*](https://www.qgis.org/en/site/forusers/download.html)*).*

*
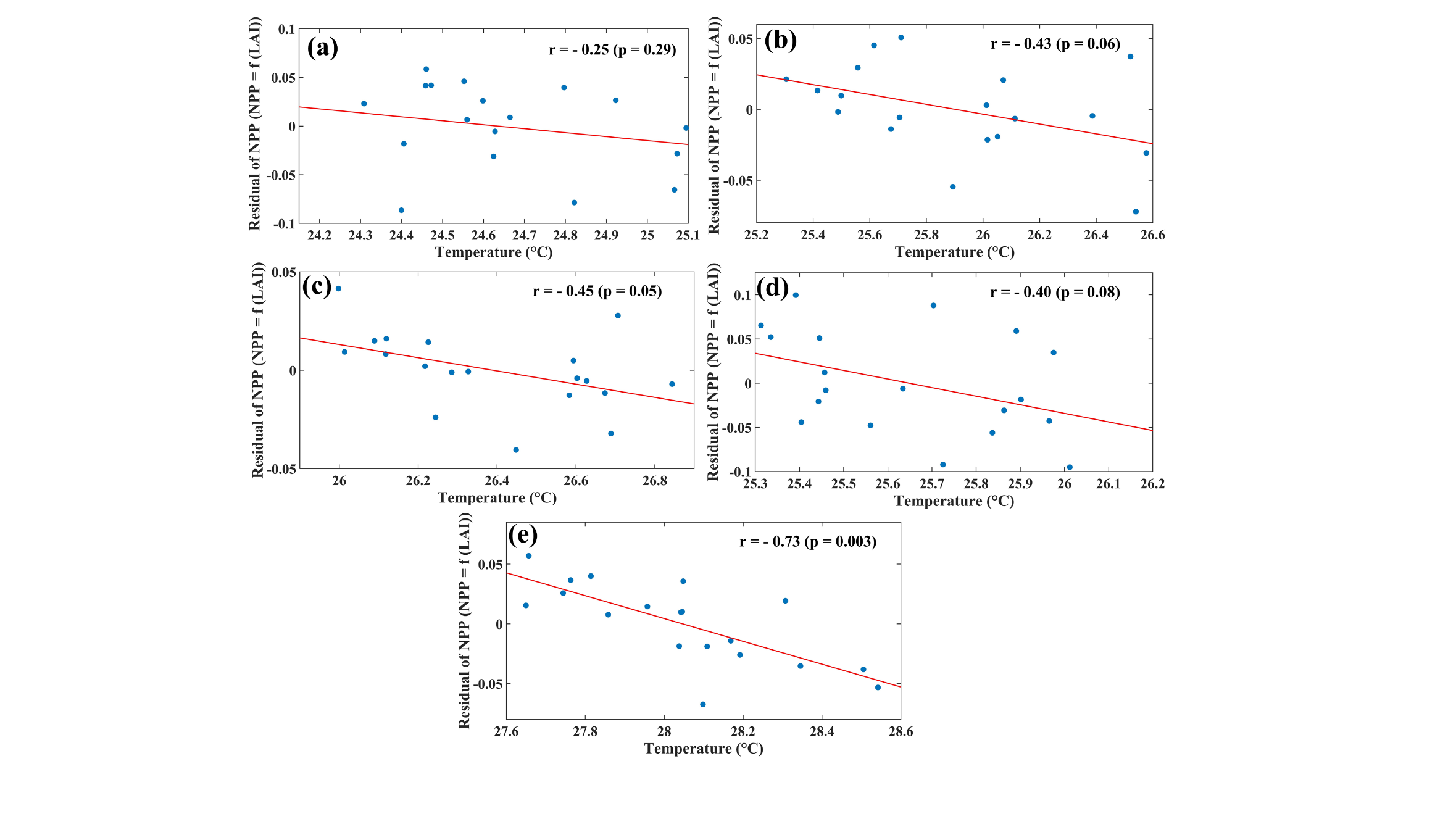
*

***Fig. S21. Impacts of Temperature on NPP:*** *Scatter plot between annual averaged temperature and residual of linearly regressed NPP as a function of annual LAI (NPP = f (LAI)) for Regions for 1(a), 2(b), 3(c), 4(d), 5 (e) during 2001-2019. Figure S21 is generated using MATLAB 2023a software (https://www.mathworks.com/?s_tid=mlh_gn_logo).*


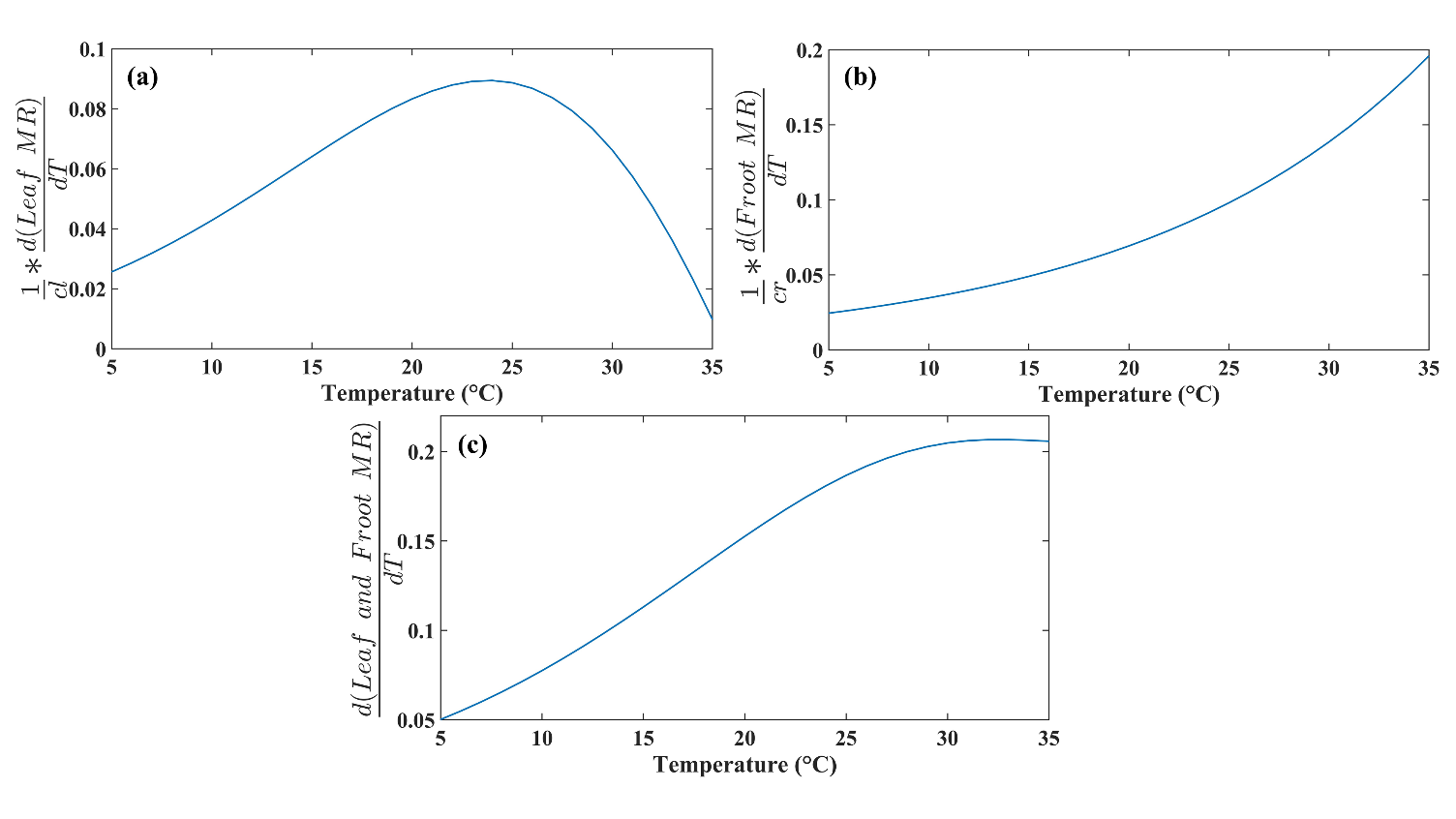


***Figure S22. Temperature sensitivity of Leaf and Fine root Maintenance Respiration:*** *Sensitivity of* $\frac{\boldsymbol{1}}{\boldsymbol{cl}}\boldsymbol{\times}\frac{\boldsymbol{d(Leaf MR)}}{\boldsymbol{dT}}$ *(a) ,* $\frac{\boldsymbol{1}}{\boldsymbol{cr}}\boldsymbol{\times}\frac{\boldsymbol{d(Froot MR)}}{\boldsymbol{dT}}$ *(b) and* $\frac{\boldsymbol{d(Leaf and Froot MR}}{\boldsymbol{dT}}$ *(c) to temperature. Figure S22 is generated using MATLAB 2023a software (https://www.mathworks.com/?s_tid=mlh_gn_logo).*


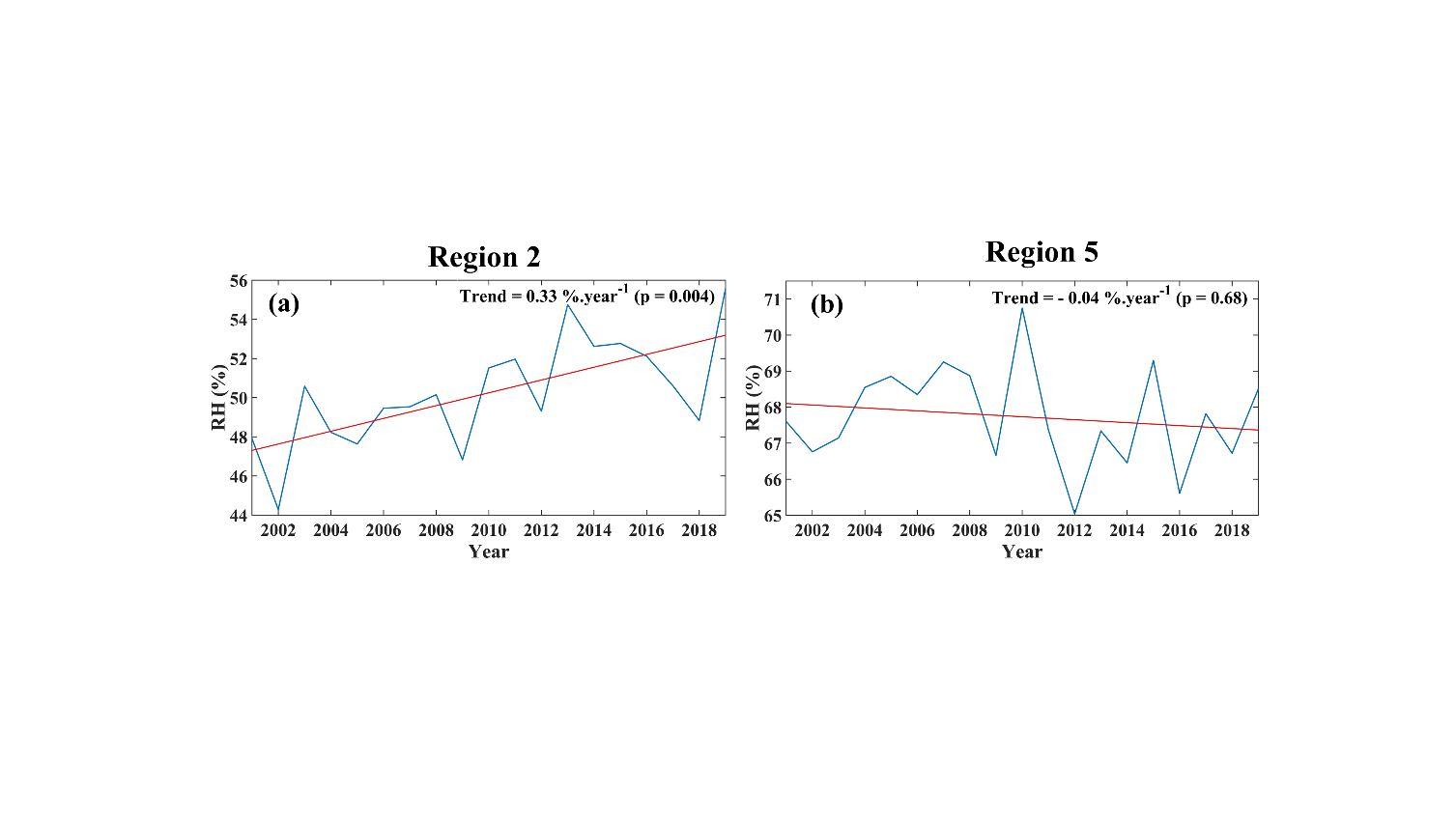


***Figure S23. Changes in Humidity****: Time series of Relative Humidity (%) over Region 2 (a) and Region 5 (b) during the period 2001-2019. Figure S23 is generated using MATLAB 2023a software (https://www.mathworks.com/?s_tid=mlh_gn_logo).*


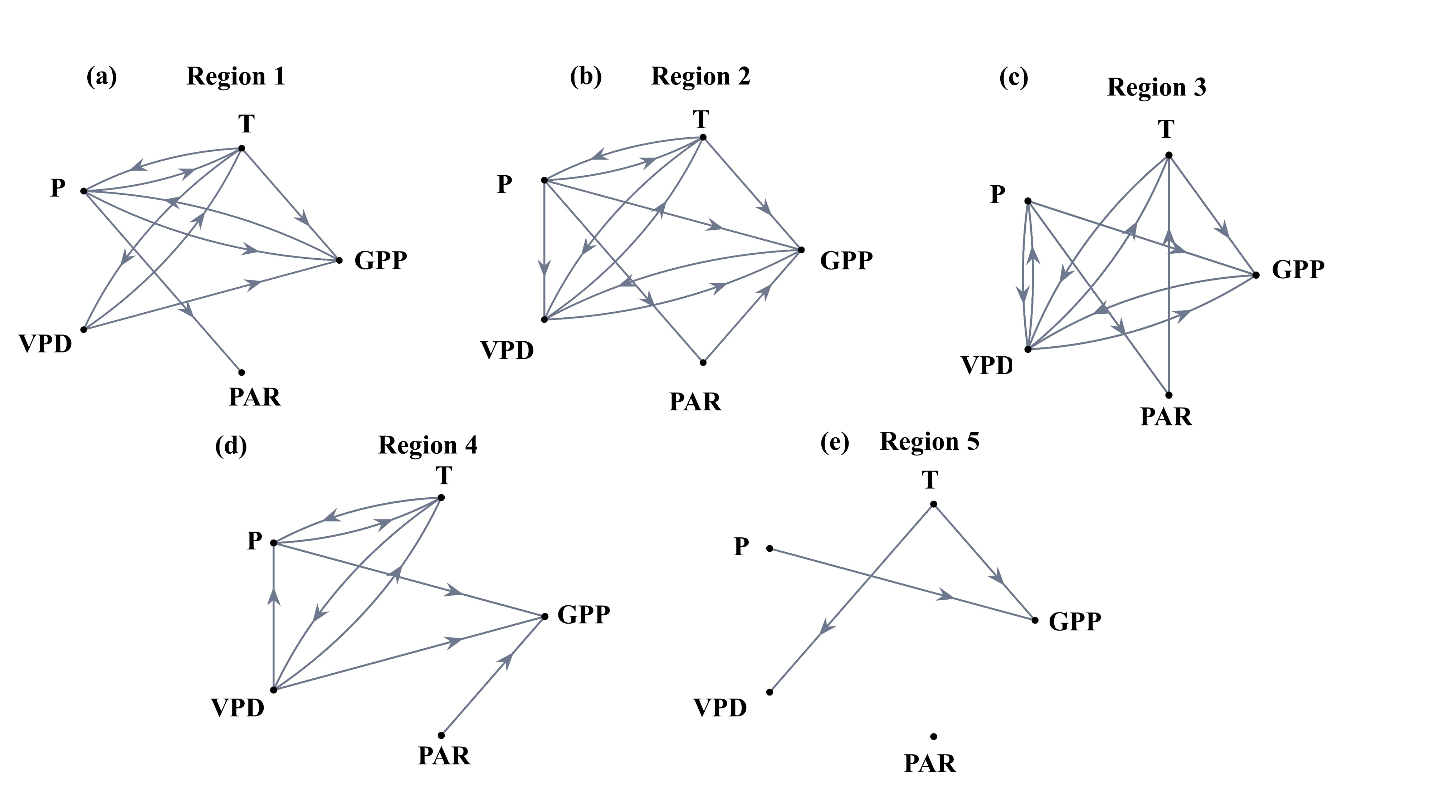


***Figure S24. Climate impacting GPP in India:*** *The causal network derived from Granger causality in the Regions 1 (a), 2(b), 3(c), 4(d), 5 (e). The variables used are Precipitation (P), Temperature (T), Vapor Pressure Deficit (VPD), Photosynthetic Active Radiation (PAR), and Gross Primary Productivity (GPP) for the period 2001-2019. The causal links are from source to sink shown using an arrow at statistically significance level 0.05. Figure S24 is generated using MATLAB 2023a software (https://www.mathworks.com/?s_tid=mlh_gn_logo).*


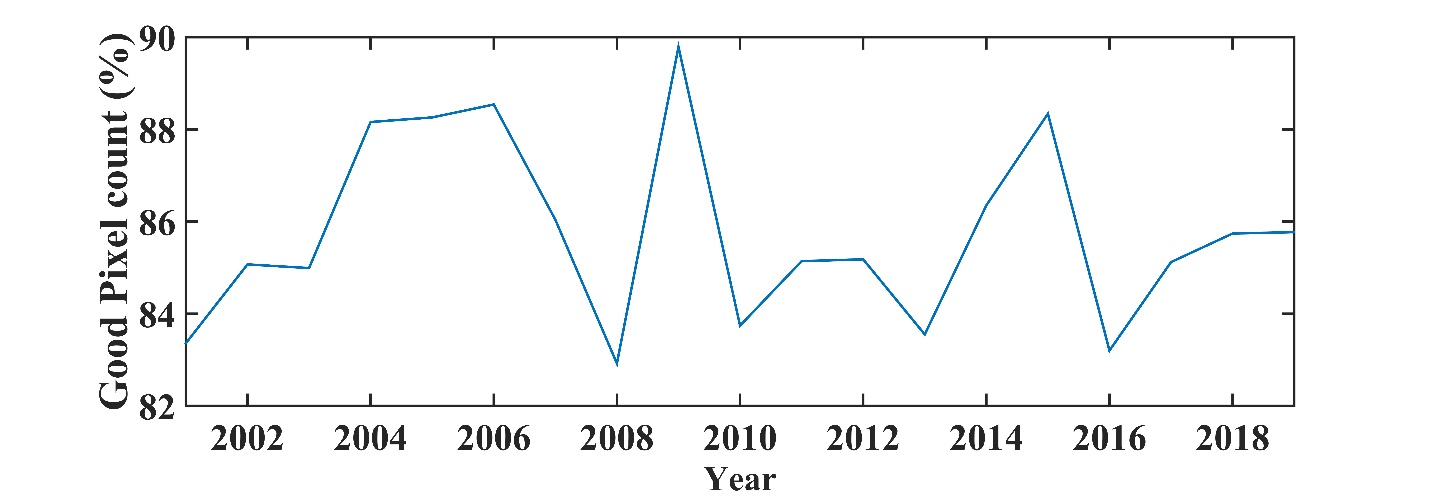


*Figure S25. Unvaried Sample size: Annual average good pixel (percentage) count over India during 2001 to 2019. Figure S25 is generated using MATLAB 2023a software (https://www.mathworks.com/?s_tid=mlh_gn_logo).*

Supplementary Text 1:

The standardization of forest cover, cropland area, LAI, and NPP brings them to the same scale, allowing us to better understand their changes in different Regions. In Region 1, we observed a sharp decline in forest cover (Fig. S15a) from the year 2017. This is due to major deforestation at isolated locations in the eastern states, such as Meghalaya, Arunachal Pradesh, and Nagaland^1^. However, we could not find such a decline in NPP (Fig. S15b) over this Region. In Region 1, the cropland area also declined from 2012; however, we did not observe such a steady decline in the LAI and NPP (Fig. S15b), The interannual variability of LAI and NPP did not depend on the changes in land use and land cover (LULC) patterns over Region 1. In Region 2 (Fig. S15c) and 3 (Fig. S15e), we observed that both cropland and forest land have increased. LAI and NPP also showed steady increases in these two regions (Fig. S15d and Fig.15f). In Region 4, we observed a steady decrease in cropland and an increase in forest land (Fig. S15g). However, we do not observe any steady decrease or increase in LAI or NPP over this Region. There exist interannual variabilities in both LAI and NPP in Region 4 (Fig. S15h) with LAI showing a significant increasing trend, and NPP showing a declining trend. In Region 5, we observed that forest cover (Fig. S15i) declined from 2010 to 2016. Cropland showed a steady increase (Fig. S15i). LAI showed an increasing trend (statistically insignificant), whereas NPP showed a significant declining trend (Fig. S15j). In Region 5, we observed an increase in Forest land cover in the year 2017 and 2019, but a continuous decrease in NPP.

Supplementary Text 2:

Analytical solution of MODIS Leaf and Fine root Maintenance Respiration equation:

We have looked into the equations for calculating MODIS NPP^1^ and solved them analytically to investigate the effect of climate variables (mainly temperature) on net primary productivity. We have mainly focused on maintenance respiration since it is highly controlled by temperature.

 MODIS NPP is calculated by the difference between GPP and Autotrophic Respiration (RA). The Autotrophic Respiration is a summation of Growth Respiration (RG) and Maintenance Respiration (RM). In the MODIS algorithm, RM = 0.25*NPP^2^.

*NPP = GPP – RA (RG+RM) Equation 1*

*NPP = 0.8 (GPP – RM), When (GPP – RM) > 0 Equation 2*

*NPP = 0 When (GPP – RM) < 0*

The Maintenance Respiration term is the summation of leaf, fine root and deadwood respiration. The Leaf Maintenance Respiration (Leaf MR) is calculated using *Equation 3*

$Leaf MR=Leaf_{mass}\times Leaf_{mrbase}\times{Q_{10}}^{\frac{T_{avg}-20}{10}}$ $Equation 3$

$Q_{10}=3.22-0.046\times T_{avg}$ $Equation 4$

$Leaf MR=Leaf_{mass}\times Leaf_{mrbase}\times\left( 3.22-0.046\times T_{avg} \right)^{\frac{T_{avg}-20}{10}}$ *Equation* 5

We substitute , 3.22 = *a* , 0.046 = *b* and$Leaf_{mass}\times Leaf_{mrbase}=cl$ and differentiate *Equation* 5 with respect to T. *Equation* 1-5 are taken from MODIS user guide ^2^.

$$\frac{d(Leaf MR)}{dT}=cl\times\left( a-b\times T_{avg} \right)^{\frac{T_{avg}-20}{10}}\times\left( \left( \frac{ln(a-b\times T_{avg})}{10} \right)-\left( \frac{-b\times\left( T_{avg}-20 \right)}{10\times\left( a-b\times T_{avg} \right)} \right) \right) Equation 6$$

$Froot MR=Fine_{rootmass}\times froot_{mrbase}\times{Q_{10}}^{\frac{T_{avg}-20}{10}}$ *Equation* 7

$Froot MR=cr\times{Q_{10}}^{\frac{T_{avg}-20}{10}}$ *Equation* 8

Froot MR is the Fine root Maintenance Respiration. In the MODIS algorithm, Q_10_ is constant value 2.0 for Froot^2^. We substitute$Fine_{rootmass}\times froot_{mrbase}=cr$ and differentiate *Equation* 8 with respect to T

$\frac{d(Froot MR)}{dT}=cr\times{Q_{10}}^{\frac{T_{avg}-20}{10}}\times\frac{\ln\left( Q_{10} \right)}{10}$ *Equation* 9

Putting the values of a, b in *Equation* 6 and Q_10_ in *Equation* 9. We have plotted the temperature response of $\frac{1}{cl}\times\frac{d(Leaf MR)}{dT}$ and $\frac{1}{cr}\times\frac{d(Froot MR)}{dT}$ separately in the Figure S18 (a) and (b) respectively.

We discovered that initially, Leaf MR rate increased with increasing temperature (Figure S18a), but after a certain temperature limit, it began to fall. The temperature limit where Leaf MR rates fall will vary depending on biome type. We have taken cl as constant since it is not a function of temperature. However, the Froot MR rate keeps on increasing with an increase in temperature (Figure S18b). The rate of increase will vary across biome types but will always be positive since cr > 0. We have taken cr as constant since it is not a function of temperature.

For analyzing the collective effect of both leaf and froot MR rate changes with temperature, we have taken

$\frac{d(Leaf and Froot MR)}{dT} = \frac{1}{cl}\times\frac{d(Leaf MR)}{dT}+ \frac{1}{cr}\times\frac{d(Froot MR)}{dT}$*Equation* 10

We have plotted the temperature response of $\frac{d(Leaf and Froot MR)}{dT}$ in the Supplementary Fig 18c. We can observe that, initially, leaf and respiration rates increase with an increase in temperature, but after a certain temperature, they remain stable. The temperature at which the rate of change will be stable will also vary across different biome type.

***Supplementary Table 1:*** *Significance level (p-value) for each causal link presented in the Granger causality diagrams for the Regions 1 (a), 2(b), 3(c), 4(d), 5 (e). The variables used are net Photosynthesis (PSNnet), Temperature (T), Precipitation (P), Vapor Pressure Deficit (VPD), and Photosynthetic Active Radiation (PAR), The rows are the Source and columns are the sink.*

| **S T** | **Region 1** | | | | |  |  | **Region 2** | | | | |
| --- | --- | --- | --- | --- | --- | --- | --- | --- | --- | --- | --- | --- |
|  | **PSNnet** | **T** | **P** | **VPD** | **PAR** |  |  | **PSNnet** | **T** | **P** | **VPD** | **PAR** |
| **PSNnet** | X | 0.09 | 0.008 | 0.84 | 0.81 |  | **PSNnet** | X | 0.53 | 0.09 | 0.12 | 0.70 |
| **T** | 0.004 | X | 6.80*10^-12^ | 5.00*10^-15^ | 0.11 |  | **T** | 0.2215 | X | 0.007 | 0.00000039 | 0.64 |
| **P** | 0.0004 | 0.000023 | X | 0.35 | 0.03 |  | **P** | 0.0001 | 0.04 | X | 0.32 | 0.69 |
| **VPD** | 0.01 | 0.0012 | 0.34 | X | 0.48 |  | **VPD** | 0.032 | 0.000244 | 0.55 | X | 0.29 |
| **PAR** | 0.99 | 0.91 | 0.73 | 0.46 | X |  | **PAR** | 0.06 | 0.06 | 0.81 | 0.77 | X |
|  | **Region 3** | | | | |  |  | **Region 4** | | | | |
|  | **PSNnet** | **T** | **P** | **VPD** | **PAR** |  |  | **PSNnet** | **T** | **P** | **VPD** | **PAR** |
| **PSNnet** | X | 0.30 | 0.52 | 0.04 | 0.24 |  | **PSNnet** | X | 0.63 | 0.39 | 0.64 | 0.12 |
| **T** | 0.0472 | X | 0.09 | 0.00000007 | 0.13 |  | **T** | 0.0047 | X | 0.0000009 | 0.01 | 0.13 |
| **P** | 0.0003 | 0.11 | X | 0.000102 | 0.000036 |  | **P** | 0.0219 | 0.002 | X | 0.41 | 0.13 |
| **VPD** | 0.008 | 0.000028 | 0.04 | X | 0.44 |  | **VPD** | 0.000003 | 0.0017 | 0.00951 | X | 0.26 |
| **PAR** | 0.049 | 0.04 | 0.76 | 0.11 | X |  | **PAR** | 0.02 | 0.14 | 0.76 | 0.11 | X |
|  | **Region 5** | | | | |  |  |  |  |  |  |  |
|  | **PSNnet** | **T** | **P** | **VPD** | **PAR** |  |  |  |  |  |  |  |
| **PSNnet** | X | 0.37 | 0.18 | 0.53 | 0.14 |  |  |  |  |  |  |  |
| **T** | 0.0025 | X | 0.11 | 0.01 | 0.18 |  |  |  |  |  |  |  |
| **P** | 0.0058 | 0.34 | X | 0.23 | 0.82 |  |  |  |  |  |  |  |
| **VPD** | 0.25 | 0.60 | 0.91 | X | 0.58 |  |  |  |  |  |  |  |
| **PAR** | 0.37 | 0.42 | 0.38907 | 0.69 | X |  |  |  |  |  |  |  |

***Supplementary Table 2:*** *Significance level (p-value) for each causal link presented in the Granger causality diagrams for the Regions 1 (a), 2(b), 3(c), 4(d), 5 (e). The variables used are Gross Primary Productivity (GPP), Temperature (T), Precipitation (P), Vapor Pressure Deficit (VPD), and Photosynthetic Active Radiation (PAR). The rows are the Source and columns are the sink.*

| **S T** | **Region 1** | | | | |  |  | **Region 2** | | | | |
| --- | --- | --- | --- | --- | --- | --- | --- | --- | --- | --- | --- | --- |
|  | **GPP** | **T** | **P** | **VPD** | **PAR** |  |  | **GPP** | **T** | **P** | **VPD** | **PAR** |
| **GPP** | X | 0.55 | 0.024 | 0.78 | 0.91 |  | **GPP** | X | 0.29 | 0.11 | 0.03 | 0.71 |
| **T** | 0.0250 | X | 4.23*10^-11^ | 1.56*10^-15^ | 0.11 |  | **T** | 0.01 | X | 0.0059 | 0.000007 | 0.60 |
| **P** | 0.0003 | 0.0000038 | X | 0.39 | 0.03 |  | **P** | 0.000000021 | 0.03 | X | 0.04 | 0.02 |
| **VPD** | 0.0120 | 0.002 | 0.5 | X | 0.54 |  | **VPD** | 0.006 | 0.0006 | 0.43 | X | 0.32 |
| **PAR** | 0.6010 | 0.94 | 0.94 | 0.52 | X |  | **PAR** | 0.02 | 0.0505 | 0.81 | 0.53 | X |
|  | **Region 3** | | | | |  |  | **Region 4** | | | | |
|  | **GPP** | **T** | **P** | **VPD** | **PAR** |  |  | **GPP** | **T** | **P** | **VPD** | **PAR** |
| **GPP** | X | 0.16 | 0.75 | 0.64 | 0.07 |  | **GPP** | X | 0.16 | 0.19 | 0.64 | 0.09 |
| **T** | 0.0004 | X | 0.18 | 0.000027 | 0.47 |  | **T** | 0.43 | X | 0.0000007 | 0.001 | 0.24 |
| **P** | 0.0003 | 0.415 | X | 0.00027 | 0.00022 |  | **P** | 0.0133 | 0.009 | X | 0.15 | 0.14 |
| **VPD** | 0.0008 | 0.00009 | 0.04 | X | 0.22 |  | **VPD** | 0.00001 | 0.005 | 0.01 | X | 0.22 |
| **PAR** | 0.041 | 0.03 | 0.77 | 0.59 | X |  | **PAR** | 0.009 | 0.24 | 0.64 | 0.11 | X |
|  | **Region 5** | | | | |  |  |  |  |  |  |  |
|  | **GPP** | **T** | **P** | **VPD** | **PAR** |  |  |  |  |  |  |  |
| **GPP** | X | 0.41 | 0.39 | 0.58 | 0.07 |  |  |  |  |  |  |  |
| **T** | 0.01 | X | 0.052 | 0.001 | 0.21 |  |  |  |  |  |  |  |
| **P** | 0.0015 | 0.25 | X | 0.16 | 0.82 |  |  |  |  |  |  |  |
| **VPD** | 0.12 | 0.48 | 0.93 | X | 0.45 |  |  |  |  |  |  |  |
| **PAR** | 0.97 | 0.40 | 0.42 | 0.76277 | X |  |  |  |  |  |  |  |

**References:**

1. Forest Survey of India. *Forest Cover: India State of Forest Report 2019 Volume 1 (Chapter 2)*. https://fsi.nic.in/isfr19/vol1/chapter2.pdf (2019).

2. Running, S., Mu, Q., Zhao, M. & Moreno, A. MOD17A3HGF MODIS/Terra Net Primary Production Gap-Filled Yearly L4 Global 500 m SIN Grid V006 [Data set]. NASA EOSDIS Land Processes DAAC. (2019) doi:https://doi.org/10.5067/MODIS/MOD17A3HGF.006.
